# Fruit-Specific Promoters in Plants: Advances, Regulatory Mechanisms and Applications in Plant Biotechnology

**DOI:** 10.3390/plants15152338

**Published:** 2026-07-29

**Authors:** Jinzhu Fan, Xinyi Tang, Aoxue Wang, Liguo Zhang, Mingfang Feng

**Affiliations:** 1 College of Life Sciences, Northeast Agricultural University, Harbin 150030, China; 17548215078@163.com (J.F.); 15289494018@163.com (X.T.); 2 Institute of Industrial Crops, Heilongjiang Academy of Agricultural Sciences, Harbin 150086, China; 3 College of Horticulture, Northeast Agricultural University, Harbin 150030, China

**Keywords:** fruit-specific promoters (FSPs), cis-acting elements, transcriptional regulatory network, CRISPR/Cas9, fruit molecular breeding

## Abstract

Fruit-specific promoters (FSPs) are a class of regulatory DNA sequences that direct transgene expression exclusively in fruit tissues or during specific stages of fruit development. They are indispensable molecular tools in modern agricultural biotechnology, with broad applications in fruit quality improvement, nutritional enhancement, and shelf-life extension. By functioning as precise molecular switches that regulate fruit-specific gene expression, FSPs overcome the limitations of constitutive promoters and facilitate precision molecular breeding for fruit quality improvement. This review systematically summarizes recent advances in FSP research. First, based on their spatiotemporal expression patterns, FSPs are classified into four categories: immature fruit-specific, fruit ripening-specific, whole fruit development stage-specific, and dual-stage (immature fruit/ripening) promoters. Their origins, expression characteristics, and key cis-regulatory elements are comprehensively summarized. Second, the complex transcriptional regulatory network governing FSP activity is discussed from the perspectives of cis-regulatory elements, major transcription factor families (such as *MADS*-box and *NAC* proteins), and epigenetic regulation, including DNA methylation and histone modifications. Furthermore, recent advances in key methodologies, including promoter cloning, functional characterization, and CRISPR/Cas9-mediated precise editing of cis-regulatory elements, are reviewed, together with their applications in crop genetic improvement, plant molecular farming, and fundamental molecular biology research. Finally, this review highlights the major challenges limiting the application of FSPs, including the relatively weak transcriptional activity of natural promoters, insufficient tissue specificity, and limited cross-species applicability. Future perspectives are discussed, emphasizing the integration of artificial intelligence-assisted promoter design, high-throughput screening, and single-cell omics technologies to develop finely tunable synthetic promoters. These advances are expected to provide both a theoretical foundation and technical support for precision molecular breeding in fruit crops.

## 1. Introduction

A promoter is a DNA regulatory sequence located upstream of a gene’s transcription start site that functions as a molecular switch controlling gene expression. By interacting with RNA polymerase and multiple transcription factors, promoters precisely regulate the timing, level, and tissue specificity of transcription initiation, thereby determining when, where, and to what extent a gene is expressed. As such, promoters are indispensable regulatory elements for achieving precise and controllable improvement of agronomic traits through genetic engineering.

In plant genetic engineering, promoter selection has gradually shifted from constitutive promoters to tissue-specific promoters. Although constitutive promoters, such as the Cauliflower mosaic virus 35S (*CaMV 35S*) promoter, offer the advantages of stable and robust expression and ease of application, their continuous activation of transgene expression throughout all tissues and developmental stages may result in unnecessary energy consumption, disruption of normal plant metabolism, and potential biosafety concerns. In contrast, fruit-specific promoters have attracted considerable attention for fruit quality improvement because of their high spatiotemporal specificity, efficient utilization of metabolic resources, and improved biosafety. These promoters activate downstream genes exclusively in fruit tissues or at specific stages of fruit development, thereby directing the plant’s metabolic resources toward fruit quality formation while minimizing the accumulation of transgene-derived proteins in non-edible tissues. Previous studies have demonstrated that the use of fruit-specific promoters to regulate genes involved in lycopene biosynthesis, ethylene signaling, or cell wall degradation enables the precise improvement of fruit traits, including color, flavor, and shelf life, without adversely affecting overall plant growth and development [1]. Therefore, the development and application of fruit-specific promoters provide an effective strategy for precision molecular breeding by balancing genetic engineering efficiency with biosafety, and hold considerable promise for advancing modern agricultural biotechnology.

To ensure the transparency and reproducibility of this review, a structured literature search and screening strategy was adopted. Literature searches were conducted in the Web of Science, PubMed, Scopus, Google Scholar, CNKI, and Wanfang Data databases, covering publications from database inception to July 2026. The search strategy included the following keyword combinations: (1) “fruit-specific promoter” OR “fruit-specific expression”; (2) “fruit development” OR “fruit ripening”; (3) “cis-acting element” OR “cis-regulatory element”; (4) “promoter cloning” OR “promoter activity”; and (5) “synthetic promoter” OR “promoter engineering”. Studies were included if they (1) investigated the isolation, characterization, or application of fruit-specific promoters; (2) provided experimental validation; and (3) were published in peer-reviewed journals. Studies based solely on bioinformatic predictions, publications in languages other than English or Chinese, and duplicate reports were excluded. Eligible studies were first screened by title and abstract, followed by full-text assessment. Additional relevant publications were identified through backward citation tracking of the reference lists.

## 2. Biological Basis of Promoters

### 2.1. Basic Structure and Function of Promoters

A promoter is a DNA regulatory sequence located upstream of the transcription start site (TSS) of a gene [2]. Although it does not encode proteins, its primary biological function is to recruit RNA polymerase and transcription factors, thereby initiating transcription and regulating the spatiotemporal patterns of gene expression [3]. Functionally, promoters serve as molecular switches and central regulatory hubs that control gene transcription [4].

**Figure 1:**
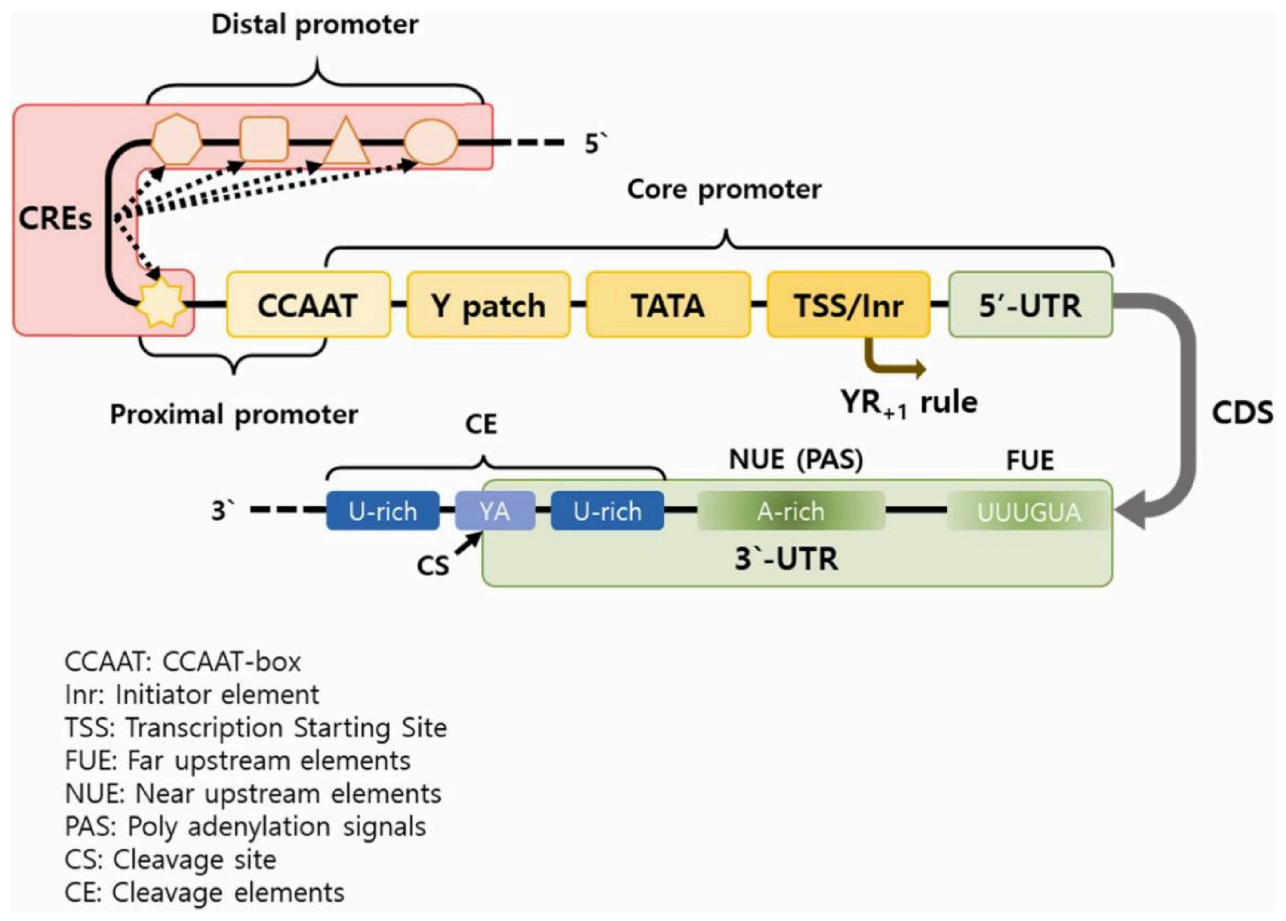
Basic structure of promoters [5]. The 5′ regulatory region comprises the distal promoter (containing conserved cis-regulatory elements), the proximal promoter (harboring the CAAT-box, Y patch, TATA-box, and Initiator element at the transcription start site), and the 5′-untranslated region (5′-UTR). In the diagram, different shapes in the distal promoter represent distinct cis-regulatory elements (CREs), and the black dashed arrows indicate that distal CREs can act on the downstream proximal promoter over a distance to achieve transcriptional activation or repression.The gene body includes the coding sequence (CDS), and the 3′ region contains the 3′-UTR with polyadenylation signal-associated elements (NUE, FUE, and downstream motifs). The cleavage site (CS) for pre-mRNA processing is also indicated. Key cis-acting elements involved in transcription initiation, promoter activity, and mRNA 3′-end maturation are integrated in this diagram.

Promoters exhibit highly sophisticated and structurally diverse architectures, which are generally divided into three functional regions: the core promoter, the proximal promoter, and the distal regulatory region. These regions act coordinately to fine-tune the level and specificity of gene expression at the transcriptional level. The structural organization of a typical promoter, as summarized by Koo H., is illustrated in Figure 1 [5].

#### 2.1.1. Core Promoter Region

The core promoter is the fundamental functional unit of a promoter and is indispensable for the transcription of all RNA polymerase II-dependent genes [6]. Located approximately 30–50 bp upstream of the TSS, it serves as the primary platform for RNA polymerase II recruitment and transcription initiation [7]. The core promoter typically contains several conserved cis-regulatory elements, including the TATA-box, Initiator (Inr), Downstream Promoter Element (DPE), and pyrimidine-rich motifs [8].

The TATA-box was the first core promoter element to be identified and is typically located 25–30 bp upstream of the TSS. It is an AT-rich conserved motif (consensus sequence: TATAAAA) that is specifically recognized by the TATA-binding protein (TBP), a subunit of the transcription factor TFIID, thereby inducing local DNA bending. Subsequently, TFIIB interacts with the adjacent DNA to stabilize the pre-initiation complex (PIC) and facilitate transcription initiation. Although the TATA-box plays a critical role in transcription initiation, it is not absolutely required for PIC assembly. In its absence, the TBP-associated factors (TAFs) within TFIID can recognize the Inr or DPE to compensate for the lack of a TATA-box and ensure efficient transcription initiation [2,9,10]. The TATA-box is also one of the most evolutionarily conserved promoter elements in both plants and animals [11].

The Inr element [8] serves as a central regulatory element in TATA-less promoters and can direct transcription initiation either independently or in cooperation with the TATA-box, thereby ensuring accurate transcription initiation [12]. The DPE is a conserved cis-regulatory element located downstream of the TSS, typically between +28 and +34 bp, with the consensus sequence RGWYV. It primarily functions by recruiting the TFIID complex, thereby promoting the assembly and stabilization of the transcription initiation complex and facilitating RNA polymerase II recruitment and efficient transcription [13,14]. Within the core promoter, the DPE acts cooperatively with the TATA-box and Inr to form the central regulatory module controlling transcription initiation. Pyrimidine-rich motifs are TC-rich sequences located upstream of the TSS that contribute to stable gene expression [15].

Collectively, the core promoter is indispensable for transcription initiation. It determines the precise position and direction of transcription initiation, integrates multiple regulatory signals, and provides the structural basis for alternative transcription initiation and bidirectional transcription.

Notably, the composition of core promoter elements varies considerably among plant species. In *Arabidopsis thaliana*, approximately 30% of protein-coding genes contain a TATA-box [6], whereas non-TATA promoters constitute the majority. In contrast, in rice (*Oryza sativa*), only approximately 18% of protein-coding genes possess a TATA-box, whereas the Y Patch is present in nearly 50% of genes, and DPE-like and BRE-like elements occur much less frequently [6]. These interspecific differences suggest that the functional conservation of core promoter elements may be lower than expected when promoters are transferred across species.

In addition to element composition, the spacing between the TATA-box, Inr, and DPE is a critical determinant of transcription initiation efficiency and promoter strength. The TATA-box is typically located 25–30 bp upstream of the TSS, and transcription initiation directed by the TATA-box generally occurs 25–30 bp downstream of this element [16]. The Inr spans the transcription start site (+1), and Inr-dependent transcription is usually initiated at the adenosine residue located at the +1 position [16]. The DPE is typically located 28–32 bp downstream of the TSS and requires strict spacing relative to the Inr for optimal activity [14]. Notably, the TATA-box and the Inr/DPE module are functionally distinct. TATA-box-containing promoters can initiate transcription in the absence of an Inr, whereas DPE-dependent promoters require a functional Inr [14]. Therefore, both the composition and spacing of core promoter elements directly determine promoter strength and transcriptional specificity and should be carefully considered in the functional design and engineering of fruit-specific promoters.

#### 2.1.2. Proximal Promoter Region

The proximal promoter is a critical cis-regulatory region that integrates endogenous and environmental signals to precisely regulate gene expression [4]. It is typically located approximately 50–200 bp upstream of the core promoter and contains a variety of cis-regulatory elements, including the CAAT-box, GC-box, and numerous signal-responsive elements (REs) [17]. These REs are associated with specific signaling pathways and include hormone-responsive elements, such as the Ethylene Response Element (ERE), Abscisic Acid Response Element (ABRE), and Gibberellin Response Element (GARE), as well as light-responsive elements (e.g., G-box and MRE) and stress-responsive elements [18].

The composition of these cis-regulatory elements determines the responsiveness of promoters to endogenous and environmental cues [19]. They regulate gene expression either independently or through cooperative interactions with distal regulatory regions [17,20]. Among the major upstream regulatory elements, the CAAT-box and GC-box are the most extensively characterized and generally function as transcriptional activators. The CAAT-box is typically located 70–80 bp upstream of the Inr element and commonly possesses the consensus sequence GCCAAT. The GC-box is usually distributed between −30 and −110 bp relative to the TSS. As a GC-rich motif, it can serve as an alternative regulatory element in TATA-less promoters and contributes to transcription initiation [21]. Together, these elements primarily regulate the efficiency and frequency of transcription initiation.

Signal-responsive elements are widely distributed within promoter regions and determine the basal transcriptional activity, tissue specificity, and transcriptional responsiveness of target genes. By serving as binding sites for specific transcription factors, they fine-tune gene expression in response to developmental and environmental signals, thereby ensuring the accuracy and flexibility of transcriptional regulation [22,23,24].

#### 2.1.3. Distal Regulatory Region

The distal regulatory region is located farther upstream of the TSS and comprises three major classes of cis-regulatory elements: enhancers, silencers, and insulators. Enhancers are long-range cis-regulatory DNA elements that regulate the spatiotemporal specificity of gene expression and substantially increase promoter activity [25,26]. In contrast, silencers function as negative regulatory elements that repress promoter activity by modulating gene expression and interfering with the binding or activity of transcription factors [27]. Insulators serve primarily as boundary elements that prevent enhancers from ectopically activating neighboring genes, thereby ensuring the specificity of gene expression and maintaining the fidelity of regulatory signaling [28].

In plants, enhancer–promoter communication is mediated through multiple regulatory mechanisms. First, enhancer activity is constrained by genomic distance. Unlike animal enhancers, which can regulate promoters located tens of kilobases away, plant enhancers are typically positioned within 1–5 kb of their target genes, and increasing distance markedly reduces their regulatory efficiency [29]. Second, chromatin looping represents a central mechanism underlying enhancer–promoter communication. Through chromatin loop formation, distal enhancers are brought into close spatial proximity to their target promoters, thereby enabling long-range transcriptional regulation. This process is mediated by the Mediator complex and cohesin proteins [30]. As a transcriptional coactivator complex, Mediator physically links transcription factors bound to enhancers with the RNA polymerase II transcriptional machinery through subunits such as *MED25*, thereby stabilizing enhancer–promoter interactions and coordinating transcription initiation [31].

These regulatory mechanisms have been experimentally validated during tomato fruit ripening. For the *E8* gene, a 428 bp promoter fragment is sufficient to confer ethylene responsiveness; however, its activity is orientation-dependent. The forward orientation confers ethylene responsiveness, whereas the reverse orientation results in elevated expression in immature fruit. At least three regulatory regions within the 5′ upstream sequence and one region downstream of the transcription unit collectively contribute to *E8* expression during fruit ripening. Multiple cis-regulatory elements, including ethylene-responsive and fruit development-related elements, act cooperatively to confer ripening-specific expression [32].

For the Polygalacturonase gene(*PG*), promoter fragments of 1.4 kb and 532 bp are sufficient to confer ripening-specific expression, whereas a 2356 bp promoter fragment reduces transcriptional activity. These findings indicate that the *PG* promoter contains both positive and negative regulatory regions that coordinately control the spatial expression pattern and transcriptional level of *PG* during fruit development [33].

For the *SlACS2* gene, the *NAC* transcription factor *SlNAP2* directly binds to the *SlACS2* promoter and activates its transcription, thereby promoting ethylene biosynthesis and fruit ripening [34]. In addition, *SlACS2* expression is positively regulated through an ethylene-mediated feedback loop. Its promoter integrates ethylene-responsive cis-elements and transcription factor-binding sites to achieve precise regulation of ethylene biosynthesis during fruit ripening.

### 2.2. Classification of Fruit-Specific Promoters

The conserved promoter architecture, consisting of the core, proximal, and distal regulatory regions, provides the fundamental framework for gene transcription [5]. However, whether a promoter exhibits fruit-specific activity is primarily determined by the unique combination of cis-regulatory elements within the proximal promoter together with the coordinated actions of enhancers and silencers in the distal regulatory region [29]. Fruit-specific promoters are typically enriched in hormone-responsive elements associated with fruit development and ripening, such as ethylene- and abscisic acid (ABA)-responsive elements, while their precise spatiotemporal expression patterns are further refined through distal regulatory elements [18].

Plant promoters are generally classified into four major categories according to their expression patterns: constitutive, tissue-specific, inducible, and synthetic promoters [35]. Fruit-specific promoters represent a specialized subgroup of tissue-specific promoters. Because their activities differ markedly throughout fruit development—from fruit set and cell expansion to the breaker and fully ripe stages [36]—a more refined classification is required to accommodate different genetic engineering applications. Accordingly, we propose four functional categories: (1) green fruit-specific promoters, which are predominantly active during early fruit development and decline in activity during ripening [37]; (2) fruit ripening-specific promoters, which are specifically activated at the breaker and red-ripe stages [33,38]; (3) promoters active throughout fruit development, which maintain continuous transcriptional activity from young fruit to full maturity [39]; and (4) green fruit–ripening dual-stage promoters, which exhibit biphasic activity during both the green fruit and ripening stages, with relatively low activity during intermediate developmental stages [40,41].

These four categories exhibit distinct regulatory characteristics. Green fruit-specific promoters are enriched in hormone-responsive elements associated with early fruit development, whereas ripening-specific promoters preferentially contain ethylene- and ABA-responsive elements. Promoters active throughout fruit development possess balanced combinations of cis-regulatory elements that support sustained transcription, whereas dual-stage promoters integrate regulatory modules associated with both early and late developmental signals, resulting in biphasic expression patterns. It should be emphasized that this classification is intended as a practical framework for promoter selection in molecular breeding rather than implying that these four categories represent mutually exclusive molecular mechanisms [16].

In addition to their functional classification, fruit-specific promoters can also be categorized according to their origin as either natural or synthetic promoters. Synthetic promoters are generally generated by multimerization of functional cis-regulatory elements or modular assembly strategies, enabling more precise spatial and temporal regulation of transgene expression.

The activities of these four classes of fruit-specific promoters have been extensively evaluated using reporter gene assays. Hiwasa-Tanase et al. identified two novel tomato promoters, *LA22CD07* and *LesAffx.6852.1.S1_at*, which exhibited strong activity in both immature green and red-ripe fruits, weak activity in flowers, and no detectable activity in other tissues. Unlike the well-characterized ripening-specific promoter *E8*, these promoters were already highly active during the green fruit stage, indicating their suitability for driving transgene expression throughout fruit development from the green to ripening stages [41]. Agarwal et al. systematically characterized the tomato *RIP1* promoter and demonstrated that β-glucuronidase (GUS) activity was highest in red-ripe fruits but remained weak in flower buds, confirming its fruit-preferential and ripening-specific expression pattern [42]. Similarly, Chung et al. reported that the *SlAdh2* promoter remained active from the appearance of young green fruits (approximately 1 cm in diameter) until full ripening, indicating its suitability for sustained transgene expression throughout fruit development [43].

The temporal responsiveness of fruit-specific promoters has also been investigated. Analysis of the tomato *Lehsp23.8* promoter showed that heat shock-induced *GUS* activity gradually increased within 48 h after treatment and subsequently declined during fruit ripening. The optimal temperatures for maximal promoter activity differed among developmental stages, reaching 42 °C in green fruits, 36 °C at the breaker stage, and 39 °C in both pink and red-ripe fruits, demonstrating that the inducible response of this promoter is strongly development-stage dependent [44].

Biphasic expression has likewise been reported for several fruit-specific promoters. Van Haaren and Houck demonstrated that the tomato *2A11* promoter exhibits high expression during fruit ripening while also showing transient activity in green fruits approximately 2–3 cm in diameter. Promoter fragments of 4.0 kb and 1.3 kb conferred strong fruit-specific *GUS* expression, whereas a 1.8 kb fragment failed to drive detectable *GUS* activity, suggesting the presence of critical negative regulatory elements within the deleted region [45]. Similarly, the *PFF* promoter characterized by Estornell et al. displays a typical biphasic expression pattern, with strong activity in the columella and placenta during early fruit development, reduced activity during intermediate developmental stages, and reactivation at the breaker stage [39].

Differences in promoter strength have also been quantitatively evaluated. Kim et al. reported that *GUS* activity driven by the *SlHDC-A* promoter in transgenic tomato fruits was approximately tenfold higher than that driven by the constitutive *CaMV 35S* promoter, demonstrating the exceptionally high transcriptional activity of this fruit-specific promoter [46]. More recently, Deng et al. employed a massively parallel reporter assay (MPRA) to identify 2436 functional enhancers across three stages of tomato fruit development (mature green, breaker, and red-ripe). A subset of these enhancers remained highly active throughout fruit development, providing a comprehensive resource for the quantitative engineering of fruit-specific gene expression [47].

#### 2.2.1. Green Fruit-Specific Promoters

Green fruit-specific promoters are a subclass of tissue-specific promoters that exhibit developmental stage-specific activity during fruit development. Their primary function is to drive the expression of downstream genes specifically during the immature green fruit stage, while their transcriptional activity is markedly reduced or completely abolished as fruits undergo ripening and coloration. These promoters generally regulate key physiological processes during early fruit development and are frequently associated with the expression of genes involved in fruit growth, pigment initiation, and secondary metabolite biosynthesis.

Among the best-characterized green fruit-specific promoters, the *TFM7* promoter exhibits high transcriptional activity during the immature green fruit stage, with its activity declining sharply upon the onset of fruit ripening [48]. Chen et al. [49] employed the *TFM7* promoter to drive fruit-specific expression of *AtCKX2* in tomato (*Solanum lycopersicum*), thereby reducing cytokinin levels before the onset of ripening. This strategy enabled a systematic investigation of the effects of cytokinin depletion on subsequent fruit ripening.

Consistent with the developmental specificity of *TFM7*, several additional promoters exhibiting high activity during early fruit development have been identified in tomato, providing valuable molecular tools for the precise regulation of gene expression during the green fruit stage. Estornell et al. [39] developed a tomato fruit-specific expression vector toolkit (pENFRUIT) based on the multisite Gateway cloning system, incorporating six newly isolated fruit-specific promoters. Among these, the *PLE* promoter is preferentially expressed during the green fruit stage, with maximal activity during early fruit expansion, whereas the *SlPPC2* promoter exhibits strong specificity during the fruit cell expansion phase.

Guillet et al. [37] performed a detailed functional analysis of the *SlPPC2* promoter and demonstrated that its 1966 bp upstream region, including the 5′ untranslated region (5′UTR) intron, was sufficient to drive strong and specific reporter gene expression during fruit cell expansion (approximately 8–25 days after anthesis, DAA), while exhibiting negligible activity in vegetative tissues such as leaves and seedlings. Further analyses revealed that promoter activity depends on the distal regulatory region (−1528 to −430 bp), whereas the 5′UTR intron functions as a negative regulatory element that is essential for maintaining developmental stage specificity. In addition, the promoter responds to the ethylene precursor 1-aminocyclopropane-1-carboxylic acid (ACC) and sugar analogs, suggesting that it integrates hormonal and metabolic signals to regulate gene expression during fruit development.

In addition to the promoters described above, the tomato *SlEXP1* promoter [50] also regulates genes associated with cell wall loosening during the green fruit expansion stage. Similar promoters exhibiting green fruit-specific or early fruit development-specific expression have also been identified in other horticultural crops. In grape (*Vitis vinifera*), the *VvAdh1* gene is highly expressed during the early stages of berry development, and its promoter has been proposed as a useful regulatory sequence for driving transgene expression in developing berries [51]. Likewise, the *MdRbcS* promoter from apple (*Malus domestica*), which regulates the ribulose-1,5-bisphosphate carboxylase small subunit gene, has been characterized as a green tissue-specific promoter. In combination with its corresponding terminator, it provides an effective regulatory cassette for high-level transgene expression in green tissues [52].

Green fruit-specific promoters therefore represent valuable molecular tools for plant genetic engineering by enabling precise trait improvement during early fruit development and targeted expression of genes associated with stress tolerance and disease resistance. Restricting transgene expression to the green fruit stage minimizes undesirable effects on fruit quality during ripening, thereby helping to preserve the commercial value and nutritional quality of the harvested fruit.

#### 2.2.2. Fruit Ripening-Specific Promoters

Fruit ripening-specific promoters are a class of promoters that are specifically activated upon the onset of fruit ripening, such as during the breaker and red-ripe stages, while exhibiting extremely low or undetectable activity in immature fruits and vegetative tissues. These promoters are typically induced by ripening-associated hormonal signals, including ethylene in climacteric fruits and ABA in non-climacteric fruits, and are enriched in corresponding hormone-responsive cis-regulatory elements, such as the ERE and ABRE. Representative examples include the *E4* [38], *E8* [53], and *PG* [54] promoters from tomato, which are among the best-characterized fruit ripening-specific promoters. Their transcriptional activity increases markedly during the breaker stage, reaches a maximum in fully ripe fruits, and is positively regulated by ethylene signaling.

Dasgupta et al. [55] identified a series of fruit-specific or fruit-preferential promoters from citrus (*Citrus* spp.) and Japanese plum (*Prunus salicina*) through bioinformatic screening followed by experimental validation. These included *CitSEP*, *CitWAX*, C*itJuSac*, *CitVO1*, and *CitUNK* from citrus, as well as *PamMybA* from plum. Functional analyses in transgenic tomato plants demonstrated distinct expression patterns among these promoters. The *CitSEP* promoter exhibited strict fruit specificity, with expression detected exclusively in ripe fruits, whereas the remaining promoters primarily directed gene expression in the pericarp, septum, and pulp, with some displaying weak activity in flowers and vegetative tissues.

Agarwal et al. [42] systematically characterized the promoter of *RIP1*, a ripening-induced gene from tomato. This promoter exhibited its highest transcriptional activity in red-ripe fruits and was almost inactive in vegetative tissues, demonstrating a clear fruit-preferential expression pattern. A series of 5′ deletion constructs derived from the full-length 1450 bp promoter were fused to the *GUS* reporter gene and introduced into tomato and *A. thaliana* through stable transformation. The results demonstrated that the full-length promoter is required to maintain both maximal transcriptional activity and fruit specificity. Deletion of regions containing fruit-specific cis-regulatory elements resulted in a substantial reduction or complete loss of promoter activity. These findings establish the RIP1 promoter as a valuable molecular tool for the precise genetic regulation of fruit ripening-related traits.

In addition to the promoters described above, ripening-specific promoters from other horticultural crops have also been extensively investigated. In grape, the *VvAdh2* and *VvGrip4* genes exhibit elevated transcript levels during berry ripening, and their promoters have been proposed as useful regulatory sequences for driving transgene expression in ripening berries [51]. In banana (*Musa acuminata*), *MaExp1* is induced by ethylene treatment before the onset of ripening, and its expression progressively increases throughout fruit ripening. Accordingly, the *MaExp1* promoter has been suggested as a promising candidate for directing transgene expression during banana fruit ripening [56].

Fruit ripening-specific promoters are particularly well suited for the genetic improvement of ripening-associated traits, including the regulation of fruit softening, pigmentation, flavor development, and other quality-related characteristics. Consequently, they represent valuable molecular tools for the precise genetic manipulation of postharvest fruit quality in horticultural crops.

#### 2.2.3. Whole Fruit Development Stage Promoters

Promoters active throughout fruit development are capable of driving target gene expression continuously from early fruit development to full maturity. Unlike stage-specific promoters, their activity is not restricted to a particular developmental stage, making them well suited for applications requiring sustained transgene expression throughout the entire course of fruit development.

Estornell et al. [39] identified the *PNH*, *PSN*, and *PHD* promoters from tomato, all of which exhibited a high degree of fruit specificity. Their expression was confined to the placenta, columella, and vascular tissues, with little or no detectable transcriptional activity in non-fruit organs. Moon [57] performed a systematic functional analysis of the promoter of *PpACO1*, which encodes 1-aminocyclopropane-1-carboxylate oxidase in peach (*Prunus persica*). A series of promoter fragments ranging from 403 to 2919 bp were fused to the *GUS* reporter gene and introduced into tomato plants. The results demonstrated that the shortest 403 bp fragment was sufficient to confer fruit-specific expression but lacked responsiveness to fruit ripening. In contrast, the promoter fragment containing the −1319 to −901 bp region exhibited stronger transcriptional activity, whereas only the full-length 2919 bp promoter reproduced the progressive increase in expression from the green fruit stage to full ripening and retained ethylene responsiveness.

Based on microarray analyses of the tomato cultivar ‘Micro-Tom’, Hiwasa-Tanase et al. [41] identified two promoters, *LA22CD07* and *LesAffx.6852.1.S1_at*, that are highly expressed during fruit development. The *LA22CD07* promoter exhibited gradually increasing activity beginning at 12 days after anthesis and reached maximal expression in red-ripe fruits. In contrast, the *LesAffx.6852.1.S1_at* promoter was already active at the ovary stage and remained active throughout fruit development. Histochemical *GUS* staining further confirmed that both promoters were strongly expressed in green and ripe fruits while exhibiting only minimal activity in non-fruit tissues such as leaves and roots, demonstrating their high degree of fruit specificity.

Promoters active throughout fruit development have also been identified in other horticultural crops. In kiwifruit (*Actinidia chinensis*), the promoter of the actinidin gene regulates gene expression during fruit development, and its activity is conserved in transgenic petunia, while sequence variation within the promoter among cultivars contributes to differences in actinidin accumulation [58]. In apple (*M. domestica*), a 450 bp promoter fragment of the ACC oxidase gene successfully drove GUS expression in transgenic tomato plants. Because its activity was not restricted to the ripening stage, this promoter may be suitable for directing transgene expression throughout a broader range of fruit developmental stages [59]. Furthermore, several receptacle-specific promoters identified in woodland strawberry (*Fragaria vesca*), including *pGene06301* and *pGene19774*, exhibited transcriptional activity from early receptacle development through fruit maturity. Some promoters showed a gradual decline in activity during fruit maturation, whereas others maintained sustained expression in both the receptacle and seeds [60]. These findings demonstrate that promoters active throughout fruit development exhibit diverse regulatory characteristics across different plant species.

#### 2.2.4. Green Fruit-Ripening Dual-Stage Promoters

Fruit-specific dual-stage promoters are a class of promoters that exhibit transcriptional activity during two key phases of fruit development: the immature green fruit stage and the ripening stage. Their activity is typically reduced during intermediate developmental stages, resulting in a characteristic biphasic expression pattern. These promoters are well suited for genetic engineering strategies that require coordinated regulation of target genes during both early fruit development and fruit ripening.

The 2A11 promoter [40] was one of the earliest fruit-specific dual-stage promoters identified in tomato. It drives strong and highly specific expression of the *2A11* gene in fruit tissues, with little or no detectable activity in vegetative or other reproductive tissues. Similarly, the *PFF* promoter, isolated by Estornell et al. [39] using the tomato fruit-specific expression vector toolkit pENFRUIT, exhibits a characteristic biphasic expression pattern. It is specifically activated in the columella and placenta during the early stages of fruit development, gradually decreases in activity as the fruit matures, and is subsequently reactivated at the onset of fruit ripening.

Zhou et al. [61] employed the tomato fruit-specific *P119* promoter to drive the expression of an artificial microRNA (amiRNA) targeting the *pectate lyase* (*PL*) gene in cultivated strawberry (*Fragaria×ananassa* Duch.) The *P119* promoter efficiently directed amiRNA expression in strawberry fruits, resulting in a 34.5% reduction in *PL* transcript abundance. Fruits from the *PL*-silenced lines exhibited significantly greater firmness than those of the control plants, whereas anthocyanin accumulation was unaffected, demonstrating the potential of the *P119* promoter for precise genetic regulation without compromising fruit quality.

Promoters exhibiting biphasic or dual-stage expression have also been identified in other horticultural crops. Dasgupta et al. [55] reported that the citrus *CitWAX* and *CitJuSac* promoters displayed strong fruit-preferential expression, with high activity in fruits, weak activity in flowers, and little or no detectable expression in other tissues. In apple (*M*. *domestica*), transcriptional activity of the *MdNAC18.1* promoter is influenced by an insertion/deletion (InDel) polymorphism and is maintained during both early fruit development and fruit ripening [62]. In addition, the tomato *SlAdh2* promoter remains active from the appearance of green fruits through full ripening, indicating its suitability for driving transgene expression throughout fruit development and ripening [43]. Collectively, these findings suggest that promoters exhibiting dual-stage expression are widely distributed among horticultural crops and represent valuable molecular tools for the precise regulation of gene expression across multiple developmental stages.

A summary of representative fruit-specific promoters is provided in Table 1.

#### 2.2.5. Artificial Synthetic Promoters

To date, most fruit-specific promoters have been isolated and characterized from naturally occurring plant species. However, natural tissue-specific promoters often exhibit inherent limitations, including species-dependent activity and variable transcriptional strength [82]. For example, a fruit-specific promoter isolated from one plant species may fail to drive efficient target gene expression or exhibit unstable activity when introduced into a different species. To overcome these limitations, increasing attention has been directed toward the development of synthetic promoters. Synthetic promoters are artificially designed DNA regulatory sequences with optimized expression characteristics that are absent in nature. They are typically generated through rational design or directed evolution by combining functionally characterized cis-regulatory elements from different sources with a minimal core promoter [83].

Compared with natural promoters, synthetic promoters offer several important advantages, including tunable transcriptional strength, improved tissue specificity, higher induction efficiency, lower basal expression, and programmable responsiveness to environmental or developmental signals. These features enable precise spatial and temporal control of gene expression while minimizing metabolic burden and unintended pleiotropic effects. In addition, synthetic promoters can be used to establish orthogonal regulatory systems that function independently of endogenous regulatory networks, making them powerful tools for plant molecular breeding, synthetic biology, and plant-based biomanufacturing.

You et al. [84] designed a synthetic promoter responsive to fruit ripening by analyzing cis-regulatory elements within the promoters of target genes regulated by the tomato *ERF* transcription factor SlERF.G3-Like, successfully reproducing the ripening-specific expression pattern of native promoters. Li et al. [85] constructed the dual hormone-responsive synthetic promoter SJ-609 by combining cis-regulatory elements responsive to salicylic acid (SA) and jasmonic acid (JA). This promoter exhibited rapid and robust responses to both phytohormones as well as to multiple plant pathogens, demonstrating the feasibility of engineering customized promoters for biotic and abiotic stress-responsive regulatory systems to enhance crop disease resistance. Kim et al. [86] identified a critical 50 bp region (−626 to −577 bp) within the tomato *SlHDC-A* promoter. Through interactions with GATA, HSF, and AP1-like transcription factors, this region functions as the core regulatory module required for fruit-specific expression. Furthermore, a synthetic promoter containing three tandem copies of this 50 bp element exhibited substantially higher transcriptional activity in transgenic tomato fruits than the constitutive *CaMV 35S* promoter, providing an efficient regulatory tool for fruit-specific transgene expression.

The design of synthetic promoters generally follows a modular assembly strategy, in which functionally validated cis-regulatory elements (e.g., hormone-responsive elements, tissue-specific elements, enhancers, and silencers) are combined with a minimal core promoter, such as the minimal *CaMV 35S* promoter or a TATA-box-containing promoter [16]. Several key principles should be considered during promoter design. First, appropriate cis-regulatory elements should be selected according to the desired expression pattern. Second, the arrangement and spacing of these elements are critical for transcriptional synergy. The “grammar” of promoter architecture—including motif identity, distance from the transcription start site, spacing between motifs, helical phasing of transcription factor-binding sites, motif orientation, and combinatorial interactions among motifs—has a profound influence on transcriptional activity [4]. Third, compatibility between cis-regulatory elements and the selected core promoter should be carefully evaluated, as different core promoters can markedly influence promoter performance. Finally, tandem multimerization of key cis-regulatory elements can enhance transcriptional activity, although excessive repetition should be avoided because it may induce transcriptional repression or gene silencing.

Despite their enormous potential for precise control of gene expression, synthetic promoters still face several important challenges. First, the molecular mechanisms underlying the interactions between cis-regulatory elements and transcription factors, as well as enhancer–promoter communication, remain incompletely understood. The construction of synthetic regulatory circuits based on natural regulatory modules is therefore constrained by limited knowledge of plant gene regulatory networks, the relatively small number of well-characterized promoters, and the potential for crosstalk with endogenous regulatory pathways [87]. Second, experimentally validated cis-regulatory elements remain scarce in non-model plant species, limiting the broad application of synthetic promoters across diverse crops. Third, synthetic promoters may be aberrantly activated or repressed by endogenous transcription factors, resulting in unintended expression patterns [87]. Finally, synthetic promoters may undergo epigenetic silencing during successive generations, and their long-term genetic stability requires comprehensive evaluation. Together, these challenges highlight the need to integrate rational design with high-throughput screening strategies in future synthetic promoter development.

Recent years have witnessed substantial advances in synthetic promoter engineering. Artificial intelligence (AI)-assisted design has emerged as a powerful strategy for constructing biological parts, regulatory modules, genetic circuits, and even entire regulatory systems by integrating high-throughput biological data with machine learning approaches [88]. Deep learning-based promoter design models can identify complex combinatorial rules governing cis-regulatory motifs from large-scale sequence datasets, thereby substantially improving the efficiency of synthetic promoter design. In parallel, massively parallel reporter assays (MPRAs) enable the simultaneous functional evaluation of thousands of synthetic promoter variants in a single experiment [47]. In addition, Golden Gate-based modular cloning technologies have greatly simplified the rapid assembly, optimization, and iterative improvement of synthetic promoters. Looking forward, synthetic promoter engineering is expected to evolve toward intelligent, signal-responsive regulatory systems that integrate multiple sensory modules to coordinate gene expression in response to diverse environmental and developmental cues, thereby providing increasingly precise molecular tools for the improvement of complex agronomic traits [88].

**Figure 2:**
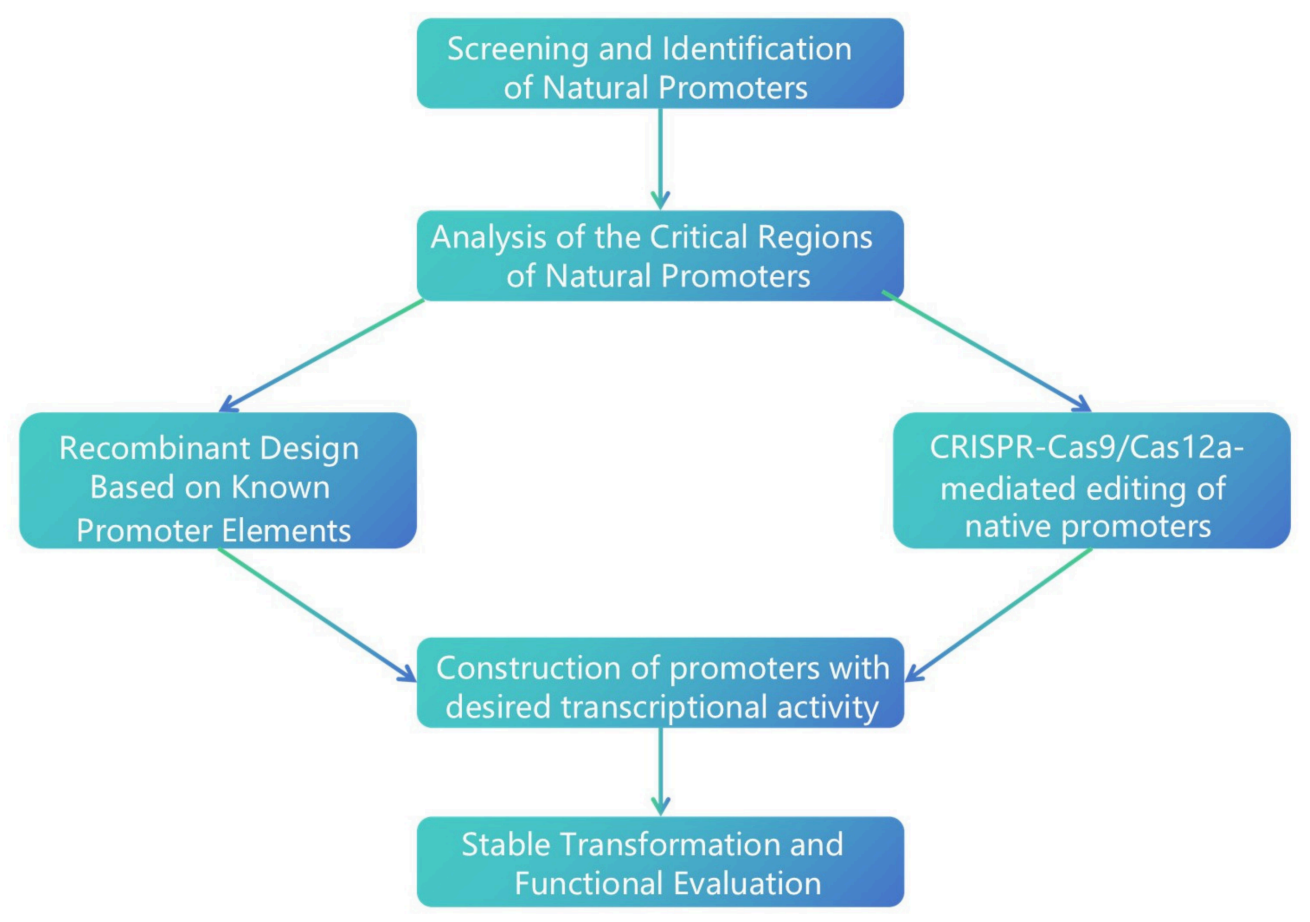
Flowchart of artificial promoter synthesis. The pipeline includes screening and identification of natural promoters, analysis of critical regulatory regions (core elements and transcription factor binding sites), followed by either recombinant design using known promoter elements or CRISPR-Cas9/Cas12a-mediated editing of endogenous promoters. Candidate promoters with desired transcriptional activities are then constructed through combinatorial approaches and validated by stable transformation and functional assays.

The general workflow for synthetic promoter design and construction is illustrated in Figure 2.

The rational design of synthetic promoters generally follows a systematic workflow. First, motif selection involves identifying key cis-regulatory elements associated with the desired expression pattern from target promoters using databases such as PlantCARE [89] and PlantPAN 3.0 [90], as well as RNA-seq and ATAC-seq datasets. Second, modular assembly integrates the selected cis-elements with a minimal core promoter, while standardized cloning platforms, such as Golden Gate and MoClo, enable their rapid and efficient assembly [91]. Third, spacer optimization fine-tunes the spacing and arrangement of cis-elements to facilitate synergistic transcription factor binding, as motif spacing, helical phasing, and motif orientation have significant effects on transcriptional activity [4]. Fourth, chromatin accessibility prediction evaluates the accessibility of the target promoter in specific cell types using ATAC-seq data or computational tools such as seq2PRINT [92]. Finally, functional validation begins with transient expression assays for rapid screening, followed by stable genetic transformation to confirm spatiotemporal expression specificity and heritable promoter activity.

## 3. Regulatory Mechanisms of Fruit-Specific Promoters

Fruit development and ripening are highly coordinated and orchestrated biological processes, involving the precise expression of hundreds of genes in a spatiotemporally specific sequence. This precision relies on a complex regulatory network within the upstream promoter region of target genes. A promoter is not a simple “gene switch” but a highly modular “information integration hub” that finely modulates the expression intensity, onset timing, and response patterns of genes in fruit tissues through the complex interplay between cis-regulatory elements (CREs) and trans-acting factors.

In recent years, with the rapid advancement of technologies such as high-throughput sequencing, gene editing, and artificial intelligence, research in this field has advanced from the identification of individual regulatory elements to the global analysis of regulatory networks and the era of artificial synthetic design. This chapter will systematically elaborate on the regulatory mechanisms of fruit-specific promoters, uncovering the molecular basis underlying their fruit-specific regulatory capacity at the levels of cis-elements, trans-acting factors, hormone-responsive elements, and epigenetic regulation.

### 3.1. Cis-Regulatory Elements of Fruit-Specific Promoters

CREs are conserved short DNA motifs located within promoter sequences. They serve as specific binding sites for transcription factors and represent the fundamental functional units governing transcriptional regulation. Sequence analyses of fruit-specific promoters have shown that their functional specificity is jointly determined by core promoter elements and tissue-specific regulatory elements. The two most important core elements are the TATA-box [5] and the CAAT-box [5]. The TATA-box plays a central role in defining the transcription start site, whereas the CAAT-box primarily enhances promoter activity and transcriptional efficiency.

In addition to these core elements, fruit-specific promoters are enriched in diverse cis-regulatory elements that respond to endogenous signals, such as phytohormones, and exogenous stimuli, including light and environmental stress. Together, these elements confer tissue specificity and developmental stage-dependent expression. For example, Li et al. [93] isolated a 1159 bp upstream promoter region of the citrus *CsPMEI-InvI* gene using genome walking. Sequence analysis identified 13 CAAT-boxes, 18 TATA-boxes, and 11 classes of cis-regulatory elements associated with light responsiveness, anaerobic induction, circadian regulation, low-temperature responsiveness, JA signaling, and SA signaling. Cai et al. [94] reported that, in pomelo (*Citrus grandis*), a single-nucleotide substitution within the core sequence of the W-box cis-regulatory element (GGTCAA to GATCAA) in the *CitSEP* promoter abolished binding of the transcriptional repressor *CgWRKY1*, thereby relieving transcriptional repression of downstream genes. In contrast, a mutation within the core sequence of the *CgGAD4* promoter (AGTGT to AATGT) enhanced the binding affinity of its transcriptional activator, ultimately promoting the fruit-specific expression of genes involved in citrate degradation.

Lin et al. [95] identified a *NAC* recognition element (consensus sequence: CACG) within the promoter of *RrGGP2* from *Rosa roxburghii* Tratt., which serves as the primary binding site for the upstream transcription factor *RrNAC56*. Under stress conditions, *RrNAC56* specifically binds to this cis-regulatory element and activates *RrGGP2* transcription, thereby enhancing ascorbic acid (AsA) biosynthesis. This study provided mechanistic insight into the molecular basis underlying the exceptionally high vitamin C accumulation in *R. roxburghii*. Similarly, in papaya (*Carica papaya*), the transcription factor *CpbZIP52* specifically binds to G-box and ABRE-like cis-regulatory elements (core sequences: ACGT and CACGTG, respectively) within the promoters of ripening-related genes, thereby activating their transcription and promoting fruit ripening [96].

Collectively, these studies demonstrate that both core promoter elements (e.g., the TATA-box and CAAT-box) and diverse signal-responsive cis-regulatory elements are indispensable for the function of fruit-specific promoters. The composition, abundance, and spatial organization of these cis-regulatory elements collectively determine promoter specificity, transcriptional activity, and responsiveness to developmental and environmental cues. Based on comparative analyses of promoters from multiple plant species, these cis-regulatory elements can be broadly classified into five major categories: Core Promoter Elements, Hormone-Responsive Elements, Light-Responsive Elements, Stress- and Defense-Responsive Elements, and Transcription Factor-Binding Sites. Among these, hormone-responsive elements integrate multiple phytohormone signaling pathways, including ABA, gibberellin (GA), auxin (IAA), methyl jasmonate (MeJA), SA, and ethylene, reflecting the complex hormonal regulation that coordinates fruit development and ripening. The consensus sequences and biological functions of representative cis-regulatory elements are summarized in Table 2.

Beyond the characterization of individual CREs, recent studies have increasingly focused on the systematic analysis of motif enrichment, motif density, spatial distribution, and combinatorial interactions within fruit-specific promoters. These studies have provided new insights into the molecular architecture underlying promoter function.

#### 3.1.1. Motif Enrichment and Combinatorial Interactions

Analysis of the promoters of the tomato *Sly-miR167* gene family revealed distinct enrichments of hormone-responsive cis-regulatory elements. The *Sly-miR167a* promoter contains two auxin-responsive elements (AuxREs), three ethylene-responsive elements (EREs), and three gibberellin-responsive elements (GAREs), whereas the *Sly-miR167b* promoter contains two EREs and one TGA element (AuxRE), and the *Sly-miR167c* promoter contains four GAREs [64]. Similarly, among tomato genes involved in fruit pigmentation, 10 of the 23 genes in the carotenoid biosynthetic pathway and 10 of the 40 genes in the flavonoid biosynthetic pathway contain both ABRE and ERE motifs within their 2 kb upstream promoter regions [107], suggesting coordinated regulation of fruit pigmentation by ABA and ethylene through combinatorial cis-regulatory element configurations. In citrus, genome-wide analyses of promoters of key carotenoid biosynthetic genes revealed that ABA-responsive motifs are abundant across all seven *Citrus* species examined, whereas ethylene-responsive motifs are largely absent, indicating a distinctive phytohormone regulatory mechanism underlying carotenoid accumulation in citrus fruits [108]. Likewise, promoter analysis of the grape *VvABCB* gene family identified abundant light-responsive cis-regulatory elements together with motifs responsive to gibberellin, auxin, and abiotic stresses [109].

#### 3.1.2. Motif Density and Spatial Distribution

The tomato *E8* promoter contains three copies of a Cys-binding element located at positions −676 to −683, −1393 to −1400, and −1457 to −1464, respectively [110]. Genome-wide analysis of transcription factor-binding sites (TFBSs) in *Arabidopsis thaliana* demonstrated that these sites are not randomly distributed but instead follow an approximately bell-shaped distribution, with the highest density occurring approximately 50 bp upstream of the TSS. Notably, 86% of TFBSs are located between −1000 bp and +200 bp relative to the TSS [111]. In tomato, a ~50 bp core region within the *SlHDC-A* promoter was identified as essential for fruit-specific expression through interactions with GATA, HSF, and AP1 transcription factors [86].

#### 3.1.3. Cooperative Interactions Among Transcription Factor-Binding Sites

Accumulating evidence indicates that transcription factor-binding sites frequently function cooperatively rather than independently. In strawberry, the *ABCC8* promoter is activated by the *MYB* and *bHLH* transcription factors *MYB10*, *bHLH33*, and *MYC1*, demonstrating functional cooperation between *MYB*-binding sites and E-box motifs in regulating anthocyanin transporter gene expression [112]. In tomato, the *MADS*-box transcription factors *FUL2* and *MADS1* form a transcriptional complex that directly binds to CArG-box motifs within the *ASMT5* promoter, thereby activating gene expression. Electrophoretic mobility shift assays (EMSAs) and dual-luciferase reporter assays confirmed both cooperative DNA binding and synergistic transcriptional activation [106]. In addition, the *NAC* transcription factor NOR-like1 has been identified as a positive regulator of tomato fruit ripening and functions within a broader transcriptional regulatory network involving *NAC* and *MADS*-box family members [34], suggesting potential cooperative regulation between NAC-binding sites and CArG-box motifs during fruit ripening.

#### 3.1.4. Large-Scale Identification of Functional Enhancers

Recent advances in high-throughput technologies have enabled genome-wide functional characterization of fruit-specific regulatory elements. Deng et al. [47] employed a massively parallel reporter assay (MPRA) to synthesize 11,180 promoter fragments derived from 1118 fruit-specific genes and quantified enhancer activity during three stages of tomato fruit development (mature green, breaker, and red-ripe) as well as in leaves. This analysis identified 2436 functional enhancers, including a subset that remained highly active throughout fruit development. In addition, fine-scale deletion analysis of a watermelon fruit-specific promoter identified two negative regulatory regions (−986 to −959 bp and −472 to −424 bp), within which the TCCAAAA motif was identified as a previously uncharacterized fruit-specific cis-regulatory element [113].

Collectively, these studies demonstrate that fruit-specific promoters possess highly diverse cis-regulatory architectures. Individual promoters frequently contain multiple hormone-responsive, light-responsive, and stress-responsive cis-regulatory elements that function cooperatively to regulate gene expression. This combinatorial organization provides the molecular basis for the precise spatiotemporal regulation of fruit-specific genes by integrating developmental signals, tissue-specific programs, and environmental cues. Core promoter elements, such as the TATA-box and CAAT-box, ensure basal transcriptional activity, whereas the composition, abundance, spatial organization, and combinatorial interactions of regulatory elements collectively determine promoter specificity, developmental stage-dependent activity, and responsiveness to environmental stimuli.

### 3.2. Trans-Acting Factors and Transcriptional Regulatory Networks

The regulatory functions of CREs depend on their recognition by specific trans-acting factors. Among these, transcription factors constitute the principal trans-acting regulators that precisely activate or repress target gene transcription by binding to specific cis-regulatory elements within promoter regions, thereby controlling gene expression in a spatially and temporally coordinated manner. Fruit development and ripening are highly coordinated biological processes that require the concerted action of multiple transcription factor families, which together form a hierarchical regulatory network.

In this section, we systematically review the major transcription factor families involved in fruit-specific gene regulation, the cis-regulatory elements they recognize, their functional roles in fruit development and ripening, and the molecular mechanisms underlying their coordinated interactions.

#### 3.2.1. Key Transcription Factor Families and Their Regulatory Functions

##### MADS-Box Family

*MADS*-box transcription factors constitute a highly conserved family of regulatory proteins that play indispensable roles in plant growth and development. All members contain a conserved *MADS* DNA-binding domain composed of approximately 56–58 amino acids [114,115]. Based on their structural characteristics, *MADS*-box proteins are classified into Type I and Type II subfamilies, with Type II proteins, also known as MIKC-type *MADS*-box transcription factors, serving as the principal regulators of reproductive development and fruit ripening. Members of this family specifically recognize CArG-box cis-regulatory elements, which are widely distributed within the promoters of ripening-associated genes and function as the primary binding sites through which *MADS*-box transcription factors regulate target gene expression [116].

**Figure 3:**
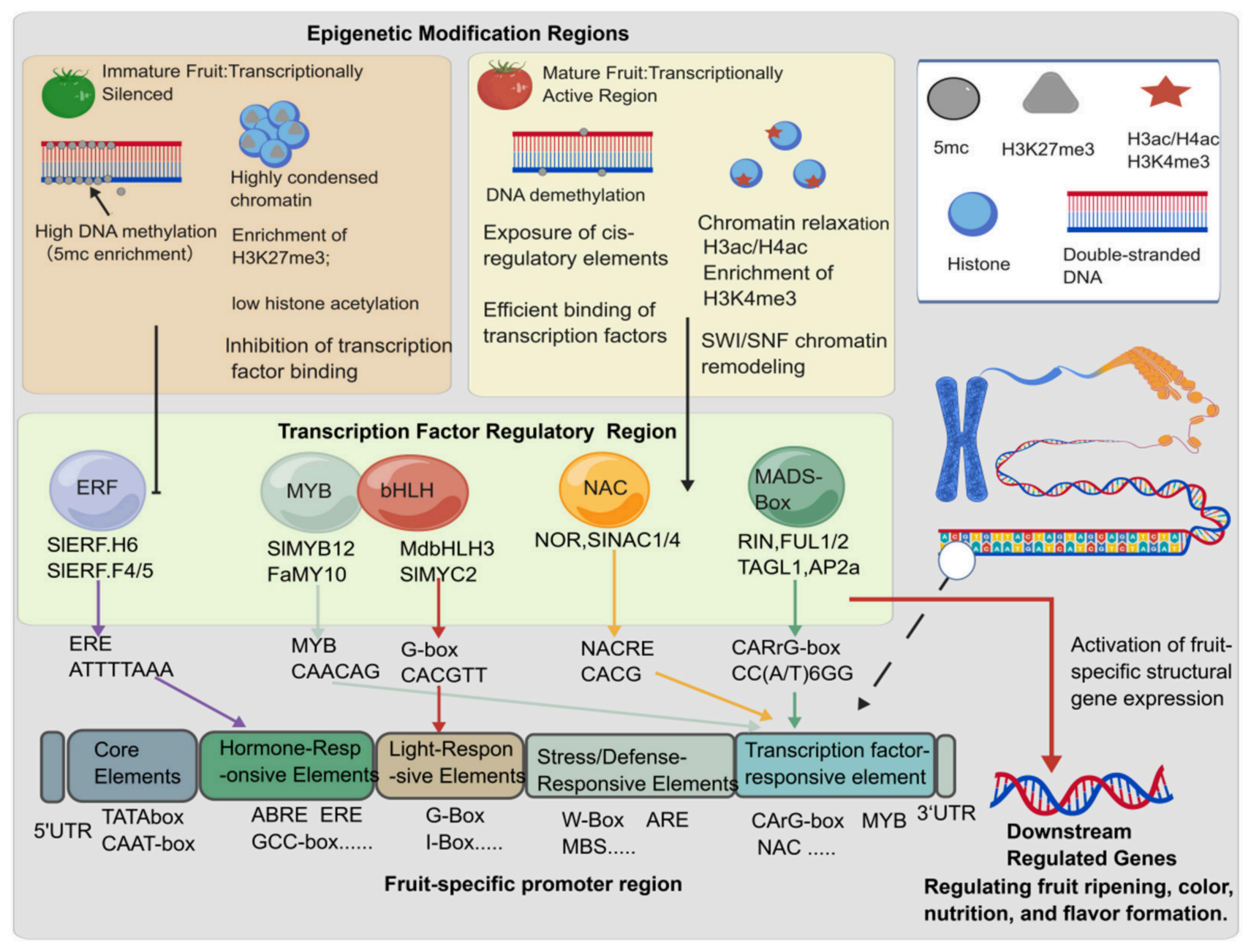
Model of integrated regulatory mechanism for fruit-specific promoters. The model is organized into three hierarchical layers. (**Upper panel**): Epigenetic regulation—in immature fruits, the chromatin is condensed, with high DNA methylation (5mC), enrichment of H3K27me3, and low histone acetylation, which collectively suppress transcription factor binding. Upon fruit maturation, DNA demethylation occurs, chromatin becomes relaxed, and cis-regulatory elements are exposed. (**Middle panel**): Transcriptional regulatory network—key transcription factor families (MADS-box, NAC, MYB, bHLH, and ERF) and their specific recognition motifs (CArG-box, NACRE, MYB motif, G-box, and ERE) are depicted. (**Lower panel**): Downstream biological outcomes—the combined effects of epigenetic remodeling and transcription factor networks modulate target gene expression, thereby influencing fruit ripening, coloration, nutritional quality, and flavor development. In the middle right part of the figure, the white circle and the black dashed line pointing to the promoter indicate a magnified view. Black arrows indicate activation, and T-bars indicate repression. Additionally, purple arrows indicate ERF-mediated activation, cyan arrows indicate MYB-mediated activation, thin red arrows indicate bHLH-mediated activation, yellow arrows indicate NAC-mediated activation, green arrows indicate MADS-box-mediated activation, and thick red arrows indicate activation of downstream gene expression. Created with BioGDP.com [168].

In climacteric fruits such as tomatoes, *MADS*-box transcription factors constitute the central regulatory hub of the fruit ripening network. Representative members include *RIN* (Ripening Inhibitor), *FUL1/FUL2* (FRUITFULL), *TAGL1* (TOMATO AGAMOUS-LIKE 1), and *AP2a*. These transcription factors coordinately activate or repress downstream ripening-related genes through binding to CArG-box motifs within their promoters (Figure 3), thereby initiating and maintaining the fruit ripening program.

Xu et al. [106] demonstrated that, in tomato fruit, the tandemly arranged *MADS*-box transcription factors *FUL2* and *MADS1* form a regulatory module that directly activates serotonin biosynthesis by binding to the CArG-box motif within the promoter of the downstream target gene *ASMT5*. During pineapple fruit ripening, Liu et al. [117] reported that the *MADS*-box transcription factor *AcAGL9* cooperates with the BEH family transcription factor *AcBEH1* to form a regulatory module that directly activates the expression of key carotenoid biosynthetic genes through binding to CArG-box motifs in their promoters, thereby promoting carotenoid accumulation.

In tomato, the master ripening regulator RIN binds to two non-canonical CArG-box motifs within the promoter of *GAME5*, thereby activating its own transcription. In addition, *RIN* acts cooperatively with *FUL1* and *FUL2* to promote the biosynthesis of the non-toxic steroidal glycoalkaloid esculeoside A, thereby positively regulating non-bitter glycoalkaloid metabolism in tomato fruit independently of the ethylene signaling pathway [118].

##### NAC Family

The *NAC* family is one of the largest families of plant-specific transcription factors, characterized by a highly conserved N-terminal *NAC* DNA-binding domain of approximately 150 amino acids [119]. *NAC* transcription factors play pivotal roles not only in fruit ripening but also in a wide range of biological processes, including plant growth and development [120], flower development [121], and responses to biotic and abiotic stresses [122]. Members of the *NAC* family primarily recognize *NAC* recognition elements (NREs) within the promoters of fruit-specific genes, thereby regulating downstream gene expression.

Among them, *NOR* (NON-RIPENING) is one of the earliest identified master regulators of tomato fruit ripening and belongs to the *NAC* transcription factor family. The nor mutant exhibits severe defects in fruit ripening; however, unlike the rin mutant, ethylene biosynthesis is only partially impaired. *NOR* physically interacts with *RIN* to form a transcriptional regulatory complex that cooperatively binds to the promoters of ripening-associated genes, thereby coordinating the fruit ripening process (Figure 3) [123].

In addition, *SlNAC*1 and *SlNAC*4 participate in tomato fruit softening by regulating the expression of genes involved in cell wall metabolism, including the *PG* and pectinesterase (*PE*) genes [50]. In *R. roxburghii*, *RrNAC56* specifically binds to the *NAC* recognition element (NRE; core sequence: CACG) within the promoter of *RrGGP2*, thereby activating its expression under stress conditions and promoting ascorbic acid (AsA) biosynthesis. This regulatory mechanism contributes to the exceptionally high vitamin C accumulation observed in *R. roxburghii* fruits [63]. These findings indicate that *NAC* transcription factors not only play central roles in regulating fruit ripening but also make important contributions to the accumulation of nutritional metabolites during fruit development.

##### MYB and bHLH Families

The *MYB* and *bHLH* transcription factor families frequently function cooperatively to regulate diverse biological processes, particularly the biosynthesis of secondary metabolites during fruit development. Members of the *MYB* family contain a highly conserved *MYB* DNA-binding domain at the N terminus, which typically comprises one to four imperfect repeats (R), each consisting of approximately 50–53 amino acids. Each repeat folds into three α-helices that together form a helix–turn–helix (HTH) structure responsible for sequence-specific DNA recognition [124]. *MYB* transcription factors specifically recognize *MYB*-binding sites (MBSs), such as the CAACAG motif, within the promoters of target genes, thereby activating or repressing gene transcription. The basic helix–loop–helix (bHLH) family represents another large group of transcription factors that are widely distributed in eukaryotes. Members of this family contain a conserved *bHLH* domain and preferentially recognize E-box and G-box cis-regulatory elements (Figure 3) [125].

In apple (*M. domestica*), anthocyanin biosynthesis in the fruit peel is regulated by a transcriptional complex formed by *MdMYB1* and *MdbHLH3*. This complex cooperatively activates the expression of key anthocyanin biosynthetic genes, including *MdDFR*, *MdUFGT*, and *MdANS*, through binding to *MYB-*binding sites (CAACTG) and E-box motifs (CACGTT) within their promoters [126]. Recent studies further demonstrated that the ABA signaling regulator *MdABI5* enhances the interaction between *MdMYB1* and *MdbHLH3* and promotes the binding of *MdbHLH3* to target promoters, thereby positively regulating ABA-induced anthocyanin accumulation [127]. These findings demonstrate that the coordinated action of *MYB*-binding sites and E-box motifs provides the molecular basis for activation of the *MYB–bHLH* regulatory complex.

In tomato, flavonoid biosynthesis in the fruit peel is primarily regulated by *SlMYB12*. *SlMYB12* activates the transcription of flavonoid biosynthetic genes, including *SlCHS* and *SlCHI*, by binding to *MYB*-binding motifs (e.g., CAACAG and ACCAACC) within their promoters, thereby determining the characteristic yellow coloration of tomato peel. More recently, *SlMYC2*, a *bHLH* transcription factor and a central component of the jasmonic acid signaling pathway, was shown to interact with the Mediator subunit *SlMED25*. This complex directly binds to the CACRYG motif, an E-box variant, within the *SlMYB12* promoter and activates its transcription by promoting histone acetylation, thereby initiating the downstream flavonoid biosynthetic pathway [128].

In strawberry (*F.×ananassa*), anthocyanin biosynthesis is positively regulated by Fa*MYB10*, whereas *FaMYB1* functions as a negative regulator. Because *FaMYB1* lacks the domain required for interaction with bHLH proteins, it competitively binds to *MYB*-binding sites (CAACTG) within the promoters of target genes, thereby preventing the recruitment of the *FaMYB10–bHLH* activation complex and consequently suppressing anthocyanin accumulation [129]. Another negative regulator, FaMYB6-like, also inhibits Fa*MYB10*-mediated anthocyanin biosynthesis [130]. Together, these findings illustrate the importance of competitive binding to *MYB*-binding elements in fine-tuning anthocyanin accumulation during strawberry fruit development.

Collectively, *MYB* and *bHLH* transcription factors primarily regulate fruit pigmentation, including anthocyanin and carotenoid biosynthesis, as well as the production of flavor-related metabolites, while also participating in plant responses to environmental stresses. In many cases, they function as part of the MBW (*MYB–bHLH–WD40*) transcriptional complex to coordinately regulate the expression of downstream target genes.

##### ERF Family

Ethylene Response Factor (*ERF*) proteins belong to the *AP2/ERF* superfamily and are characterized by a conserved *AP2/ERF* DNA-binding domain of approximately 60 amino acids [131]. *ERF* transcription factors specifically recognize EREs and GCC-box cis-regulatory elements (Figure 3), thereby playing pivotal roles in ethylene signaling, stress responses, and plant developmental regulation [132].

Climacteric fruits are characterized by a pronounced increase in respiration during ripening, known as the respiratory climacteric, which is accompanied by a burst of endogenous ethylene production. This transition marks the onset of irreversible fruit ripening and senescence and is associated with a series of physiological and biochemical changes, including color development, fruit softening, and aroma formation. In climacteric fruits such as tomato, *ERF* transcription factors function as key downstream regulators of the ethylene signaling pathway by modulating the expression of genes involved in carotenoid biosynthesis, cell wall remodeling, and volatile aroma production.

Yang et al. [133] elucidated the molecular mechanism by which oligogalacturonides (OGs), generated through the action of fungal polygalacturonase, delay tomato fruit softening. OGs activate ethylene-responsive elements within fruit-specific promoters, thereby inducing the expression of *SlERF.H6*. *SlERF.H6* subsequently cooperates with other components of the ethylene signaling pathway to directly repress the transcription of cell wall metabolism-related genes, including *SlPL* and *SlEXP1*, thereby effectively delaying fruit softening.

Liu et al. [134] further demonstrated that core ethylene-responsive cis-elements, including EREB and E-box motifs, function as key regulatory nodes within fruit-specific promoters through their interactions with *ERF* transcription factors such as *SlERF.F4* and *SlERF.H5.* On the one hand, these *ERF* proteins directly bind to ethylene-responsive elements in the promoters of ripening-associated genes, thereby activating downstream genes involved in cell wall metabolism, pigment biosynthesis, and aroma production. On the other hand, *ERFs* participate in hierarchical transcriptional regulatory networks together with upstream regulators, including *RIN* (*MADS*-box), *NAC*, and *HD-ZIP* (HB) transcription factors, thereby integrating ethylene signaling with developmental cues to ensure the precise temporal and tissue-specific regulation of fruit ripening.

Regarding the impact of InDels on promoter responsiveness, a 4 bp InDel variant (ACS2-TAAAATAT) in the apple *MdNAC25* promoter was identified as a bidirectional enhancer element. This InDel not only enhanced *MdNAC25* expression itself but also simultaneously activated the upstream antisense-transcribed lncRNA-nac25, forming an “lncRNA-NAC transcription factor” synergistic regulatory module. This finding reveals a mechanism by which InDels regulate gene expression through enhancer–promoter communication. Furthermore, molecular evolution analysis of the tomato *E8* promoter across 16 tomato accessions revealed that deletions or insertions are present in the ethylene biosynthesis functional region (−1702 to −1274) and a negative effect region (−1253 to −936). These InDel variations may affect the ethylene responsiveness and fruit ripening-specific expression of the *E8* promoter by altering the arrangement and spacing of key cis-elements within the promoter.

#### 3.2.2. Structural and Functional Diversity of Transcription Factor Binding Sites and TF Cooperativity/Competition

Different transcription factor (TF) families recognize distinct cis-regulatory elements; however, sequence variations within the same motif can markedly influence TF-binding affinity and regulatory specificity. Fruit ripening-related gene promoters are particularly enriched in such regulatory complexity.

##### CArG-Box Sequence Variation and MADS-Box Binding Specificity

The CArG-box, with the consensus sequence CCW6GG (where W = A/T), serves as the canonical binding site for the *MADS*-box transcription factors *RIN*, *FUL1*, *FUL2*, and *TAGL1* [135]. However, not all promoters containing CArG-boxes are recognized equally by *RIN*. Studies have shown that only 24 putative promoter sequences harbor *RIN*-specific CArG-boxes, indicating that subtle variations in the flanking sequences and central region of the motif can substantially influence RIN-binding efficiency [136]. Analysis of the tomato α-mannosidase (α-Man) promoter further demonstrated that three CArG-boxes within a 567 bp promoter fragment collectively mediate *RIN*-dependent transcriptional activation [137]. Structural studies have further shown that the DNA architecture of the CArG-box, particularly the presence of A-tract motifs in its central region, plays a critical role in DNA recognition by *MADS*-domain proteins. These structural features influence not only DNA-binding site recognition but also the binding specificity of different *MADS*-box family members and their heteromeric complexes [135].

##### NAC Recognition Elements and Competitive Dimerization

NAC transcription factors also exhibit considerable diversity in binding specificity and regulatory interactions. *NOR* and NOR-like1 recognize *NAC*-binding sites in the promoters of their target genes. The tomato transcription factor *RAR* (Ripening Accelerator) directly binds to the *ACS2* promoter and represses its expression. In addition, RAR forms a heterodimer with *NOR*, and this heterodimer exhibits weaker transcriptional activation than the *NOR* homodimer, indicating that RAR negatively regulates *NOR*-mediated activation [138]. This finding reveals a novel mechanism by which *NAC* transcription factors regulate the initiation of fruit ripening through competitive dimerization [138].

##### MYB/bHLH Motif Recognition and MBW Complex Formation

*MYB* transcription factors recognize *MYB*-binding sites (MBS; core sequence CAACAG), whereas *bHLH* transcription factors bind E-box (CANNTG) and G-box (CACGTG) motifs. In tomato, these proteins can assemble into *MYB–bHLH–WD40* (MBW) complexes that coordinately regulate flavonoid and anthocyanin biosynthesis [139]. However, the tomato *WD40* protein *SlAN11* interacts with *bHLH* proteins but not with *MYB* proteins, suggesting that different *MYB–bHLH* combinations exhibit distinct interaction specificities [139]. In addition to regulating secondary metabolism, *MYB* and *bHLH* transcription factors also participate in ethylene signaling and carotenoid accumulation during fruit ripening [140].

##### ERF Recognition of GCC-Box and DRE/CRT Elements

*ERF* transcription factors recognize GCC-box (core sequence AGCCGCC) and *DRE/CRT* elements. During tomato fruit development and ripening, the expression of 13 *DREB* and 19 *ERF* subfamily genes changes dynamically, and the promoters of most of these genes contain *ARF*, *DRE/CRT*, and GCC-box motifs [141]. *ERF* proteins containing *EAR* repression motifs play essential roles in balancing the activities of other *ERF* family members by competing for *DRE/CRT* and/or GCC-box binding sites in target promoters, thereby ensuring normal tomato growth and development [141].

##### Transcription Factor Cooperativity and Competitive Regulation

In addition to motif-specific recognition, cooperative and competitive interactions among transcription factors further increase the complexity of transcriptional regulation. *NOR* and *RIN* form a protein complex that cooperatively binds target gene promoters to regulate fruit ripening [123]. Similarly, *SlERF.G*3-Like and *MADS-RIN* synergistically regulate ethylene biosynthesis and the phenylpropanoid pathway [84]. In contrast, the competitive heterodimerization between *RAR* and *NOR* represents an inhibitory regulatory mechanism that attenuates *NOR*-dependent transcriptional activation [138]. Together, these cooperative and competitive interactions establish the complex transcriptional regulatory network governing fruit ripening.

Beyond sequence variation within transcription factor-binding motifs, insertion/deletion (InDel) polymorphisms also have profound effects on transcription factor binding, chromatin organization, and promoter responsiveness.

##### InDel-Mediated Regulation of Transcriptional Machinery: Effects on Transcription Factor Binding, Chromatin Accessibility, and Promoter Responsiveness

An InDel variant in the apple *MdNAC18.1* promoter has been identified as a key determinant of promoter activity. This variant alters the spatial configuration of transcription factor-binding sites, thereby affecting the recognition and binding efficiency of upstream transcription factors and ultimately regulating *MdNAC18.1* expression [62]. Similarly, detailed analysis of the tomato *KLUH* promoter demonstrated that InDel polymorphisms within specific cis-regulatory elements significantly influence transcription factor binding affinity, leading to altered downstream gene expression [142].

ATAC-seq analyses have demonstrated that promoter InDel polymorphisms alter nucleosome positioning and chromatin accessibility by modifying the physical properties of local DNA sequences, such as DNA bendability and accessibility. In tomato, naturally occurring promoter InDel variations have been associated with changes in chromatin accessibility, thereby affecting transcription factor access to their target binding sites [142].

Promoter responsiveness can also be substantially altered by InDel variation. A 4 bp InDel variant (ACS2 TAAAATAT) in the apple *MdNAC25* promoter was identified as a bidirectional enhancer element. This InDel not only enhances *MdNAC25* expression but also simultaneously activates the upstream antisense-transcribed *lncRNA nac25*, thereby forming an *lncRNA–NAC* transcription factor synergistic regulatory module [143]. This finding reveals a mechanism by which InDels regulate gene expression through enhancer–promoter communication [143]. Furthermore, molecular evolutionary analysis of the tomato *E8* promoter across 16 tomato accessions revealed insertion and deletion polymorphisms within both the ethylene-responsive functional region (−1702 to −1274) and the negative regulatory region (−1253 to −936) [110]. These InDel variations may influence the ethylene responsiveness and fruit ripening-specific expression of the *E8* promoter by altering the arrangement and spacing of key cis-regulatory elements within the promoter [110].

#### 3.2.3. Comparative Analysis of Regulatory Networks Between Climacteric and Non-Climacteric Fruits

Climacteric and non-climacteric fruits exhibit fundamental differences in respiration patterns and ethylene production, which profoundly influence their promoter architectures, transcription factor networks, and epigenetic regulatory mechanisms [144].

A major distinction between these two fruit types lies in their hormone-mediated regulatory networks. Climacteric fruits, including tomato, apple, and banana, utilize ethylene as the central regulatory hormone to initiate and coordinate ripening. Ethylene promotes autocatalytic ethylene biosynthesis through the activation of ACC synthase *(ACS*) and ACC oxidase (*ACO*) genes and triggers hierarchical transcriptional cascades mediated by *MADS*-box, *NAC*, and *ERF* transcription factor families [144]. In tomato, *MADS*-*RIN* and *NAC-NOR* function as upstream master regulators of ripening-associated ethylene signaling. These transcription factors directly bind to the promoters of ethylene biosynthesis genes, including *ACS2*, *ACS4*, and *ACO1*, thereby initiating and maintaining the ripening program [123]. Similarly, in apple, *MADS*-box transcription factors such as *MADS8/9* act as upstream regulators of ethylene biosynthesis and ripening progression [145].

In contrast, non-climacteric fruits, including strawberry, grape, and citrus, primarily rely on ABA signaling to coordinate fruit ripening processes [146]. In grape, specific *NAC* transcription factor members have been demonstrated to participate in ripening initiation, functioning mainly through ABA-dependent regulatory pathways [147]. These differences indicate that although both climacteric and non-climacteric fruits employ conserved hormone-responsive regulatory modules, the dominant hormonal signals and their downstream transcriptional cascades are distinctly organized.

The composition and hierarchy of transcription factor networks also differ substantially between climacteric and non-climacteric fruits. In climacteric fruits, the regulatory network is largely organized around *MADS*-box and *NAC* transcription factors as central regulatory hubs. *RIN* and *NOR* act as master regulators at the upper levels of the network, whereas *ERF* and *ARF* transcription factors function as downstream components that transmit ethylene-derived signals to regulate ripening-associated genes [140,148]. In tomato, *SlAP2a*, an *AP2/ERF* family member, functions as a negative regulator of ripening by balancing the activities of positive regulatory factors and modulating the expression of downstream ripening-related genes [149].

Although *MADS*-box and *NAC* transcription factors are also present in non-climacteric fruits, their regulatory hierarchies and downstream targets differ from those observed in climacteric species. In grape, *NAC* transcription factors contribute to ripening initiation but appear to function primarily through ABA-associated signaling pathways rather than ethylene-mediated regulation [147]. In strawberry, *MYB* transcription factors, particularly *FaMYB10*, serve as central regulators of anthocyanin biosynthesis, highlighting the distinct organization of transcriptional networks underlying pigmentation and ripening processes in non-climacteric fruits.

Epigenetic regulation represents another important layer contributing to the differences between climacteric and non-climacteric fruit ripening. Although both fruit types undergo dynamic changes in DNA methylation and histone modifications during development and ripening, their regulatory patterns exhibit species-specific characteristics [144].

In climacteric fruits such as tomato, the onset of ripening is accompanied by extensive genome-wide DNA demethylation, particularly within promoter regions containing CG and CHG methylation contexts. These methylation changes are further associated with reduced H3K27me3 levels and increased levels of active histone marks, including H3ac and H3K4me3, thereby facilitating the activation of ripening-related genes [150]. In non-climacteric fruits, similar epigenetic mechanisms have also been observed. For example, in pepper, CG and CHG demethylation is associated with increased chromatin accessibility and enhanced transcription factor activity during fruit development and ripening [151].

Collectively, these findings demonstrate that climacteric and non-climacteric fruits share conserved regulatory principles involving hormone signaling, transcription factor networks, and epigenetic modifications, but differ substantially in their dominant regulatory modules and molecular hierarchies. Such differences contribute to the diverse promoter architectures and expression patterns underlying fruit-specific gene regulation across plant species.

### 3.3. Regulation of Promoter Activity by Epigenetic Modifications

The preceding sections have described the regulatory mechanisms underlying the interactions between cis-elements and trans-acting factors (Figure 3). However, transcription factor recognition and binding do not occur on isolated DNA sequences; rather, they take place within the context of chromatin. In eukaryotes, genomic DNA is packaged with histone proteins to form chromatin, and chromatin accessibility, DNA methylation status, and histone modification patterns collectively establish an epigenetic landscape that influences transcriptional regulation [144]. This section focuses on the roles of epigenetic modifications, particularly DNA methylation and histone modifications, in regulating the activity of fruit-specific promoters.

#### 3.3.1. DNA Methylation Regulation

DNA methylation is an epigenetic modification involving the addition of a methyl group to cytosine residues by DNA methyltransferases, resulting in the formation of 5-methylcytosine (5mC) (Figure 3). In plants, DNA methylation occurs in three sequence contexts: CG, CHG, and CHH. Promoter-associated DNA methylation is generally correlated with transcriptional repression through multiple mechanisms, including preventing transcription factor binding to cis-regulatory elements and recruiting methyl-CpG-binding proteins that promote chromatin compaction [144,152].

The spatiotemporal expression patterns of fruit-specific genes are closely associated with dynamic changes in promoter DNA methylation during fruit development and ripening. In tomato, ripening-related genes, including the key ethylene biosynthesis genes *ACS2* and *ACS4*, as well as ethylene-responsive genes *E4* and *E8*, undergo significant reductions in promoter CG methylation levels during fruit ripening. This demethylation process is closely correlated with transcriptional activation of these genes. Notably, mutations in the ripening regulator *RIN* impair this demethylation process, suggesting a functional interaction between master transcription factors and DNA methylation-mediated regulation during fruit ripening [153,154,155].

The regulatory functions of DNA methylation in fruit development and ripening across horticultural crops have been comprehensively reviewed [156]. In citrus fruit, the methylation level of the promoter region of the carotenoid biosynthesis gene *LCYb1* is negatively correlated with fruit coloration. During ripening, promoter methylation gradually decreases, accompanied by increased gene expression [157]. This finding provides direct evidence that promoter methylation contributes to the regulatory networks controlling fruit pigmentation.

DNA methylation is also closely associated with chromatin accessibility and transcriptional regulation. In citrus fruit ripening, integrated analyses of ATAC-seq and CUT&Tag multi-omics datasets revealed that dynamic alterations in chromatin accessibility, together with DNA methylation changes, coordinately regulate the transcriptional activation of genes involved in sucrose and citric acid metabolism [158]. Similarly, studies using UV-C treatment in tomato demonstrated a close association between ATAC-seq-detected chromatin accessibility changes and DNA methylation dynamics [159]. These findings indicate that DNA methylation regulates promoter activity, at least partly, by modulating chromatin structure and influencing transcription factor accessibility to promoter cis-elements.

#### 3.3.2. Histone Modification Regulation

Histone modification represents an epigenetic regulatory mechanism that modulates chromatin structure and transcriptional activity through the addition of chemical groups (e.g., acetyl or methyl groups) to the N-terminal tails of histones [160]. Based on their effects on transcriptional regulation, histone modifications can be broadly classified into activating and repressive types. Activating modifications, such as histone acetylation (H3ac and H4ac) and H3K4me3, are generally enriched in the promoter regions of actively transcribed genes, whereas repressive modifications, including H3K9me2 and H3K27me3, accumulate at silenced loci and promote chromatin condensation [160] (Figure 3).

During tomato fruit ripening, the levels of histone H3 acetylation at the promoter regions of ripening-associated genes, including *PSY1*, *PG*, and *E4*, increase substantially [144]. Treatment with histone deacetylase inhibitors can trigger premature ripening in immature fruits, indicating that histone acetylation represents an essential epigenetic event required for the initiation of fruit ripening [161]. Tomato histone deacetylases *SlHDA1* and *SlHDA3* are highly expressed during the early stages of fruit ripening and maintain relatively low acetylation levels at the promoters of ripening-related genes, thereby preventing their premature activation. As fruit development progresses, the expression levels of these deacetylases decline, resulting in increased promoter acetylation and consequently facilitating the activation of ripening-associated genes [161,162].

In addition, the repressive histone modification H3K27me3, which is catalyzed by the Polycomb Repressive Complex 2 (PRC2), plays a critical role in maintaining tissue-specific gene repression [163] (Figure 3). In immature tomato fruits, the promoters of several ripening-associated genes, including *RIN*, *ACS2*, and *E8*, are enriched with H3K27me3 marks, maintaining these genes in a transcriptionally inactive state. Upon the onset of ripening, PRC2 activity decreases and H3K27me3 levels are reduced, allowing the coordinated activation of these genes. This epigenetic mechanism ensures that ripening-related genes remain repressed during early fruit development and are activated only at the appropriate developmental stage [164].

Histone modifications and chromatin accessibility are tightly coordinated during fruit development and ripening. In citrus fruit ripening, genome-wide profiling of H3K27ac, H3K4me3, and H3K27me3 using CUT&Tag, combined with ATAC-seq-based chromatin accessibility analysis, established a comprehensive transcriptional and epigenetic regulatory landscape of fruit ripening [158]. Activating histone marks, including H3K27ac and H3K4me3, showed extensive overlap with regions exhibiting increased chromatin accessibility, whereas the repressive mark H3K27me3 was associated with regions displaying reduced accessibility. These findings demonstrate that histone modifications regulate transcription factor accessibility and binding efficiency by modulating chromatin states.

#### 3.3.3. Other Epigenetic Regulatory Mechanisms

In addition to DNA methylation and histone modifications, chromatin remodeling and non-coding RNAs also contribute to the regulation of fruit-specific promoter activity (Figure 3). Chromatin remodeling complexes, such as SWI/SNF, regulate nucleosome positioning through ATP-dependent processes, thereby modulating the accessibility of transcription factors to promoter regions. During tomato fruit set, SNF2 family chromatin remodeling factors are significantly upregulated and participate in the transcriptional reprogramming associated with early fruit development [165].

Furthermore, non-coding RNAs, including microRNAs (miRNAs) and long non-coding RNAs (lncRNAs), can indirectly regulate promoter activity through diverse molecular mechanisms. In tomato, *lncRNA1471* regulates the expression of ripening-associated genes, including *RIN*, *NOR*, and *SlDML2*, by interacting with the ASR transcription factor, thereby negatively regulating the initiation of fruit ripening [143]. In apple, *lncRNA-nac25* and the *MdNAC25* transcription factor form a synergistic regulatory module, in which a bidirectional enhancer element simultaneously regulates the expression of both the lncRNA and its associated coding gene, thereby influencing fruit ripening progression [166]. These emerging regulatory mechanisms provide new insights into fruit-specific gene regulation; however, their underlying molecular networks and functional interactions require further investigation [156,166].

Chromatin remodeling complexes, including SWI/SNF, are closely associated with dynamic alterations in chromatin accessibility. ATAC-seq profiling enables the identification of accessible chromatin regions generated during chromatin remodeling and provides insights into the regulatory activity of chromatin remodelers. In strawberry fruit, integrated analysis of ATAC-seq and RNA-seq datasets revealed a strong association between changes in chromatin accessibility and transcriptional activation, with transcription factor-binding motifs being significantly enriched in accessible chromatin regions [167]. This proposed regulatory cascade—“chromatin remodeling → chromatin opening → transcription factor binding → gene activation”—provides evidence supporting the functional connection between epigenetic regulation and dynamic changes in transcription factor accessibility.

## 4. Research Methods and Technologies for Fruit-Specific Promoters

### 4.1. Cloning Technologies for Fruit-Specific Promoters

**Figure 4:**
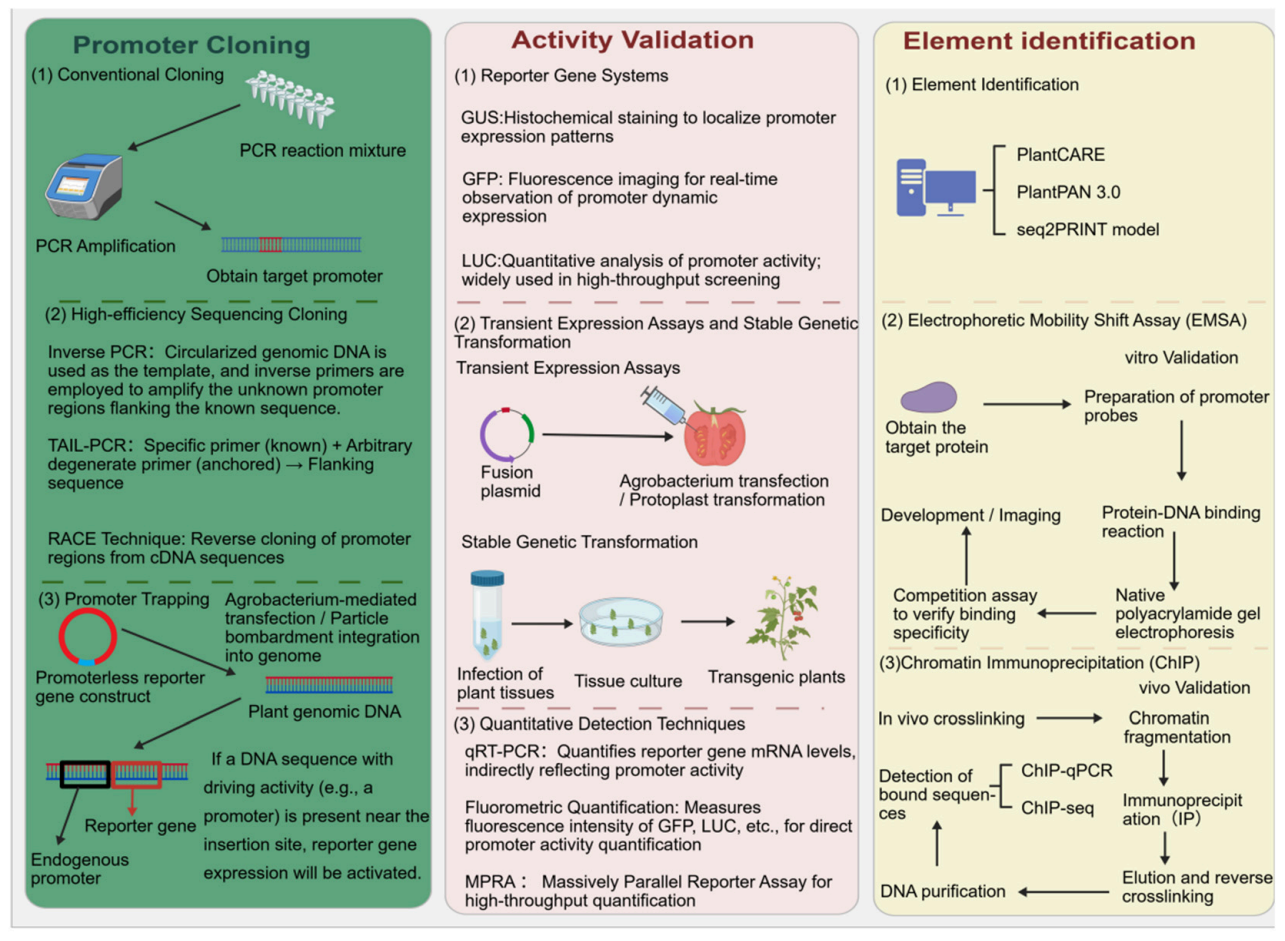
Research Methods and Technical Roadmap for Fruit-specific Promoters. The diagram comprises three interconnected sections. (**Left**) (Promoter cloning): Strategies include conventional PCR amplification from genomic DNA; high-efficiency methods such as inverse PCR, TAIL-PCR, and RACE; and promoter trapping via Agrobacterium-mediated transformation or particle bombardment. (**Middle**) (Activity validation): Approaches include reporter gene systems (*GUS*, *GFP*, *LUC*), transient expression assays, stable genetic transformation, and quantitative detection techniques (qRT-PCR, fluorometric quantification, and MPRA). (**Right**) (Element identification): Methods include bioinformatics prediction (PlantCARE, PlantPAN 3.0, seq2PRINT), in vitro validation (EMSA), and in vivo validation (ChIP-seq/ChIP-qPCR). In this diagram, black arrows indicate workflow order, material flow, and regulatory logic, linking all steps from cloning to validation to element identification. Additionally, the black and red boxes at lower left denote the promoter and exogenous gene (adjacent in DNA); the arrow below them marks these two elements. Created with BioGDP.com [168].

The cloning of fruit-specific promoters provides the foundation for their functional characterization and practical applications. With continuous technological advances, promoter cloning strategies have evolved from conventional approaches to more efficient and precise genomic-based methods. Traditional approaches mainly include PCR amplification [169] and genomic library screening. PCR amplification is suitable for cloning promoters with known sequences, offering simplicity, cost-effectiveness, and high efficiency; however, it largely depends on prior sequence information (Figure 4). In contrast, genomic library screening enables the isolation of promoters without prior sequence knowledge but is labor-intensive, technically complex, and relatively inefficient. Consequently, this approach has gradually been replaced by more advanced cloning strategies.

Efficient promoter cloning approaches mainly include inverse PCR, TAIL-PCR, and RACE-based methods, which are widely used for isolating unknown flanking sequences surrounding target genes (Figure 4). Inverse PCR involves restriction enzyme digestion of genomic DNA, followed by self-ligation of the resulting fragments and PCR amplification using primers designed based on known sequences, thereby enabling the recovery of unknown upstream regulatory regions. TAIL-PCR (Thermal Asymmetric Interlaced PCR) employs combinations of gene-specific and degenerate primers to efficiently amplify unknown genomic regions adjacent to known sequences, providing high sensitivity and specificity. RACE (Rapid Amplification of cDNA Ends) technology enables the identification of transcript ends through reverse extension from known cDNA sequences and is particularly useful for characterizing genes with complex transcriptional structures.

For example, Zhang et al. [170] focused on the *PuLOX2S* gene, which exhibits high activity during aroma compound biosynthesis in cold-stored ‘Nanguo’ pear (*Pyrus*). Using TAIL-PCR, they successfully cloned a 610 bp upstream promoter fragment and identified multiple cis-acting elements within this region. Yeast one-hybrid (Y1H) screening of a pear cDNA library subsequently identified 52 proteins capable of interacting with this promoter, including transcription factors such as *PuERF12*, *PuMYB44*, and *PuRF2a,* which may regulate *PuLOX2S* expression.

DasGupta et al. [31] successfully isolated and characterized multiple fruit-specific or fruit-preferential promoters from citrus and plum. They first identified fruit tissue-specific expressed genes through transcriptome-based screening and subsequently amplified their promoter regions using PCR-based approaches. This strategy led to the isolation of five citrus promoters (*CitSEP*, *CitWAX*, *CitJuSac*, etc.) and one plum fruit-preferential promoter (PamMybA). Fusion of these promoters with the *GUS*Plus reporter gene, followed by stable transformation into Micro-Tom tomato plants, demonstrated that the *CitSEP* promoter exhibited a strict fruit-specific expression pattern, with no detectable activity in vegetative tissues, including leaves, stems, and roots.

Promoter trapping is a functional screening strategy based on reporter gene expression and provides an alternative approach for identifying regulatory sequences. This technique involves the genomic integration of a reporter construct lacking its own promoter. When the construct is inserted near a genomic region with transcriptional regulatory activity [171], such as an endogenous promoter, the reporter gene can be activated, thereby revealing the transcriptional potential of the surrounding sequence. Furthermore, promoter trapping systems using defined promoter-reporter constructs can also facilitate the identification of regulatory elements, including enhancers, near insertion sites. Using this strategy, researchers have successfully isolated various types of promoters, including constitutive, stress-inducible, and tissue-specific promoters, in bananas [172].

### 4.2. Methods for Validating and Analyzing Fruit-Specific Promoter Activity

Validation and quantitative assessment of fruit-specific promoter activity are essential components of plant functional genomics research. Common approaches include reporter gene-based assays, transient expression analysis, stable genetic transformation, and quantitative expression analysis. These complementary strategies enable comprehensive evaluation of promoter activity, spatial expression patterns, and regulatory characteristics (Figure 4).

Reporter gene systems evaluate promoter activity by analyzing the expression of reporter genes driven by target promoters, offering highly sensitive and visually interpretable readouts. Commonly used reporter genes include *GUS*, *GFP* (green fluorescent protein), and *LUC* (luciferase). *GUS* reporter assays enable spatial localization of promoter activity through histochemical staining, *GFP* facilitates real-time visualization of promoter-driven expression using fluorescence imaging, and *LUC* provides highly sensitive quantitative measurements of promoter activity and is therefore widely applied in high-throughput screening approaches.

In studies investigating citrus phloem-specific promoters (Figure 4), Bezerra et al. [173] analyzed promoter activity by driving expression of the uidA reporter gene under the control of the *CsPP2.B1* and *CsVTE2* promoters, followed by X-Gluc histochemical staining for tissue-specific localization. For functional characterization of tomato fruit-specific promoters, *GUS* staining combined with quantitative analysis provides an effective approach for evaluating promoter activity across different fruit developmental stages. In a study of the mango *PAO* gene promoter, Bai et al. [174] employed a dual-luciferase reporter system based on the pGreenII 0800-Luc vector to quantitatively assess promoter activity associated with different deletion fragments by calculating the FLuc/RLuc ratio. The inclusion of Renilla luciferase as an internal control minimized variation caused by differences in transformation efficiency among samples. Transient expression assays in tobacco leaves further demonstrated that the −1218 to −3 bp region of the *PAO* promoter exhibited strong transcriptional activity in three different fruit tissues.

Transient expression analysis enables rapid evaluation of promoter activity through the introduction of promoter-reporter fusion constructs into plant protoplasts or via Agrobacterium-mediated transient infiltration. Due to its short experimental duration and operational simplicity, this approach is particularly suitable for preliminary promoter screening and functional validation. In contrast, stable genetic transformation provides a more comprehensive assessment by generating transgenic plants carrying promoter-reporter constructs, allowing systematic evaluation of promoter activity throughout plant development and across different tissues (Figure 4).

Yao et al. [175] developed an improved vacuum infiltration-based Agrobacterium transient expression system that enabled efficient transient transformation directly in attached tomato fruits for the first time. Using this system, they successfully identified the trichome-specific promoter AaLTPpro, providing an efficient platform for rapid screening of fruit-associated promoters. In studies investigating regulatory mechanisms of fruit-specific promoters, Yan et al. [176] employed an Agrobacterium-mediated transient expression system to demonstrate that the citrus canker pathogen TAL effectors PthA5 and PthA6 directly suppress *CsGRAS9* promoter-driven *GUS/LUC* expression, revealing a molecular mechanism by which pathogens interfere with promoter activity in fruit crops.

For stable transformation analysis, Thilmony et al. [177] generated transgenic Mexican lime plants through Agrobacterium-mediated transformation and systematically characterized the expression patterns of multiple fruit-preferential promoters. The results demonstrated that the *CitWax* promoter efficiently drove Ruby expression in fruit juice sac tissues, resulting in anthocyanin accumulation. These findings indicate that the CitWax promoter represents a useful tool for fruit-specific transgene expression, with potential applications in nutritional enhancement and improvement of fruit appearance.

Overall, transient expression assays are particularly suitable for rapid promoter screening and identification of functional regulatory elements, whereas stable transformation approaches provide a more reliable evaluation of promoter activity and spatiotemporal expression patterns at the whole-plant level. The combined application of these strategies can substantially improve the efficiency and accuracy of fruit-specific promoter characterization.

Quantitative approaches for promoter activity analysis mainly include quantitative reverse transcription PCR (qRT-PCR) and fluorescence-based assays, both of which enable accurate evaluation of promoter-driven gene expression. qRT-PCR (Figure 4) indirectly estimates promoter activity by measuring transcript abundance of reporter genes and is characterized by high sensitivity and specificity. In contrast, fluorescence-based assays directly quantify promoter activity through measurements of reporter signals, such as *GFP* fluorescence or *LUC* activity, enabling real-time and dynamic monitoring of promoter function.

Gómez-Felipe et al. [36] combined massively parallel reporter assay (MPRA) technology with a luciferase reporter system to perform high-throughput quantitative analysis of 11,800 promoter fragments derived from 1118 fruit-specific genes in tomato. Each promoter fragment was fused to a luciferase reporter gene containing a unique DNA barcode, followed by transient expression analysis in tomato fruits at three developmental stages (mature green, breaker, and red ripe) and in leaves. Based on luciferase activity measurements, 2436 functional enhancers were identified, and five synthetic enhancers exhibiting strong fruit-specific activity were subsequently designed. This study highlights the potential of luciferase-based reporter systems for high-throughput quantitative characterization of promoter activity.

Fu et al. [178] conducted a systematic investigation of the apple *MdNADP-ME* promoter. Through whole-genome resequencing and construction of a high-density genetic map, they identified stable quantitative trait loci (QTLs) associated with malate and fructose content in apple fruits and identified *MdNADP-ME* as a key candidate gene. Sequence analysis revealed that an A/C single nucleotide polymorphism (SNP) at position −799 within the promoter region affected the binding affinity of the transcription factor *MdMYB2*, thereby modulating *MdNADP-ME* expression. qRT-PCR analysis was subsequently used to quantify *MdNADP-ME* transcript levels across different apple genotypes, confirming the association between this SNP and fruit malate/fructose content. Furthermore, a dCAPS molecular marker associated with fruit fructose content was developed. This study demonstrates the importance of qRT-PCR-based expression analysis in quantitatively evaluating promoter function and linking promoter variation with fruit quality-related traits.

### 4.3. Methods for Identifying Regulatory Elements

The identification of regulatory elements is essential for elucidating the functional mechanisms underlying promoter activity. Currently, an integrated technical framework combining bioinformatics prediction with experimental validation has been well established. A commonly used strategy for promoter element analysis involves the application of databases and computational tools [93,94], such as PlantCARE and PlantPAN 3.0, to identify cis-acting elements within promoter sequences (Figure 4). These approaches enable the prediction of potential regulatory element types, positions, and distributions, thereby providing a foundation for subsequent experimental validation. Recently, the seq2PRINT model [92] was developed to directly predict transcription factor and nucleosome binding sites from DNA sequences. Compared with conventional approaches, this model exhibits substantially improved prediction accuracy and is capable of identifying the binding patterns of “cryptic” transcription factors, providing a novel computational tool for regulatory element discovery.

Electrophoretic Mobility Shift Assay (EMSA) and Chromatin Immunoprecipitation (ChIP) are widely employed approaches for validating protein–DNA interactions. EMSA detects the binding between DNA fragments and target proteins, allowing assessment of whether a transcription factor directly interacts with a specific regulatory element within a promoter region. This method is characterized by its simplicity, rapidity, and high sensitivity. In contrast, ChIP technology captures transcription factor–DNA complexes in vivo and identifies binding regions through subsequent PCR amplification or sequencing analysis. Therefore, ChIP provides a more physiologically relevant assessment of protein–DNA interactions and has become a fundamental approach for dissecting promoter regulatory mechanisms (Figure 4).

Xu et al. [76] demonstrated that the tomato *MADS*-box transcription factors *FUL2* and *MADS1* form a heterocomplex. Using ChIP-seq and EMSA approaches, they confirmed that this complex directly binds to the CArG-box motif within the *ASMT5* promoter and synergistically regulates serotonin metabolism. In strawberry fruits, DAP-seq analysis was performed to identify genome-wide binding sites of *FvSEP3*, and its direct binding to the promoters of ripening-related genes, including *FvGH3.1* and *FvNCED1*, was further validated by EMSA and ChIP-qPCR [18]. Studies on banana fruit ripening have revealed a regulatory mechanism in which redox modification modulates transcription factor activity. ChIP-qPCR and EMSA analyses demonstrated that *MaNAC42* directly binds to the promoters of oxidative stress- and ripening-related genes under oxidative stress conditions. Moreover, the DNA-binding activity of *MaNAC42* is reversibly regulated by redox modification mediated by methionine sulfoxide reductase *MaMsrB2* [179].

### 4.4. Breakthrough Applications of Cutting-Edge Technologies

The CRISPR/Cas9 system, derived from the adaptive immune mechanism of bacteria, has emerged as a core genome-editing technology for plants, animals, and microorganisms. In the field of targeted promoter engineering, this technology enables precise modification of cis-regulatory elements through sequence-specific recognition and induction of double-strand breaks (DSBs). Therefore, it provides a powerful approach for dissecting gene regulatory networks and facilitating the targeted improvement of crop agronomic traits.

In the development of fruit-specific gene-editing systems, Feder et al. [180] utilized the tomato fruit-specific *PPC2* promoter to drive Cas9 expression, successfully establishing a fruit tissue-specific gene knockout platform. In this system, the constitutive 35S promoter in the original editing vector was replaced with the *PPC2* promoter, and *GFP* was introduced as a reporter for validation. The results demonstrated that transgenic tomato plants exhibited target gene editing predominantly in fruits, whereas editing efficiency in seeds and seedlings was extremely low, consistent with the fruit-specific expression pattern of *PPC2*. Using this system to target the *SlEZ2* gene, the researchers successfully generated fruit-specific knockout lines, and subsequent phenotypic analyses revealed the important role of this gene during the late stages of fruit development.

For targeted modification of cis-regulatory elements to improve fruit yield, Li et al. [142] employed CRISPR/Cas9 technology to precisely edit a SNP located within the promoter region of the tomato *KLUH* gene, which is strongly associated with fruit weight. This SNP resides within a conserved cis-regulatory element identified by ATAC-seq analysis. The study generated 21 mutant alleles with different insertion/deletion patterns, among which five alleles consistently resulted in increased fruit weight across multiple independent evaluations. Notably, homozygous mutants m2+4bp and m3+1bp increased fruit weight by 10.7–15.7% and 8.7–16.3%, respectively. The enhanced fruit weight phenotype was consistently observed across fruits from different positions within the inflorescence.

For improving disease resistance, Jia et al. [181] generated citrus canker-resistant ‘Duncan’ grapefruit mutants through CRISPR/Cas9-mediated editing of the *LOB1* promoter. The citrus canker pathogen promotes host susceptibility by using the TAL effector PthA4 to bind the effector-binding element (EBE) within the *LOB1* promoter. Because grapefruit contains two distinct types of *LOB1* promoter sequences (TI *LOBP* and TII *LOBP*), the researchers designed two sgRNAs targeting these EBE regions and compared the editing efficiency of SpCas9p driven by the constitutive *CaMV 35S* promoter and the Yao promoter. Following Agrobacterium-mediated transformation, the biallelic mutant DunYao7 exhibited complete resistance to citrus canker, with no detectable off-target mutations.

Despite these advances, gene editing in high-ploidy fruit crops remains challenging. Sun Xiangyi et al. demonstrated in octoploid strawberry that CRISPR/Cas9-induced mutations could be partially repaired by endogenous DNA repair mechanisms, resulting in reduced editing stability. The mutation rate at the target site in the FaMYB9CR-15 line was 70.2% at three months after transformation but decreased to 43.7% at six months. Transcriptomic analysis suggested that elevated expression of genes involved in non-homologous end joining (NHEJ), microhomology-mediated end joining (MMEJ), and mismatch repair pathways may contribute to the restoration of mutated sequences [182]. These findings highlight the importance of considering endogenous repair mechanisms when designing promoter-editing strategies in polyploid fruit crops.

With the increasing application of CRISPR/Cas9 technology for fruit-specific promoter engineering, substantial progress has also been achieved in derivative technologies and biosafety assessment strategies.

For off-target analysis, whole-genome sequencing remains the gold-standard approach for identifying unintended mutations. In tomato, Slaman et al. designed 89 sgRNAs targeting members of the *MYB* transcription factor family and performed amplicon sequencing of 224 predicted off-target sites to systematically evaluate the specificity and mutation profiles of SpCas9 in tomato cells [183]. In addition, in vitro and in vivo detection approaches, including GUIDE-seq and DISCOVER-seq, have been developed to further evaluate CRISPR editing specificity. In fruit crops such as citrus and tomato, off-target assessment has become an essential component of evaluating the biosafety of genome-edited materials [183]. These approaches provide critical support for the future commercial application of fruit-specific promoter editing technologies.

For polyploid allele-specific editing, studies in octoploid strawberry have revealed the unique challenges associated with genome complexity. In CRISPR/Cas9-edited strawberry lines, the target-site mutation frequency decreased from 70.2% at three months to 43.7% at six months, suggesting that edited sequences may undergo repair by endogenous DNA repair pathways [182]. Moreover, the redundancy of homologous alleles in polyploid species requires efficient simultaneous modification of multiple alleles, which can be achieved through sgRNA design targeting conserved regions or sequential transformation strategies [184]. Systematic investigations in polyploid Brassica species by Ahmad et al. have provided valuable insights for improving CRISPR genome-editing efficiency in other polyploid crops [184].

For CRISPR activation and interference technologies (CRISPRa/CRISPRi), dCas9-based transcriptional regulation systems have been successfully applied in plant functional genomics. CRISPRa utilizes dCas9 fused with transcriptional activation domains, such as *VP64* or *EDLL*, to activate endogenous gene expression by targeting promoter regions guided by sgRNAs. In contrast, CRISPRi employs dCas9 fused with transcriptional repression domains, such as SRDX, or dCas9 alone to generate steric hindrance at promoter regions, thereby suppressing transcription [185]. In tomato, CRISPRa technology has been applied to activate fruit ripening-related genes, providing a novel strategy for regulating fruit quality traits [185].

For prime editing, the prime editor, consisting of a nickase Cas9 variant (nCas9, H840A) fused with reverse transcriptase, enables precise nucleotide substitutions, insertions, and deletions guided by prime editing guide RNA (pegRNA), without requiring donor DNA templates or inducing DSBs [186]. In tomato, the CROCS strategy employed prime editing to precisely introduce heat shock elements into the LIN5 promoter, generating heat-inducible expression patterns in tomato fruits and increasing yield by 33% under heat stress conditions [186]. The major advantage of this technology is its ability to achieve single-nucleotide-level precision in promoter modification, providing a powerful tool for fine-tuning fruit-specific promoter activity.

## 5. Applications of Fruit-Specific Promoters

### 5.1. Application in Crop Genetic Improvement

As key regulatory elements controlling gene transcription, fruit-specific promoters function as critical molecular switches in crop genetic improvement. Through targeted promoter design or modification, the expression level, spatial–temporal pattern, and stress responsiveness of target genes can be precisely regulated, enabling the controlled improvement of important agronomic traits. In recent years, strategies based on the exploitation of natural promoter variations and artificial promoter engineering have achieved significant advances in improving fruit quality and stress tolerance in horticultural crops.

**Figure 5:**
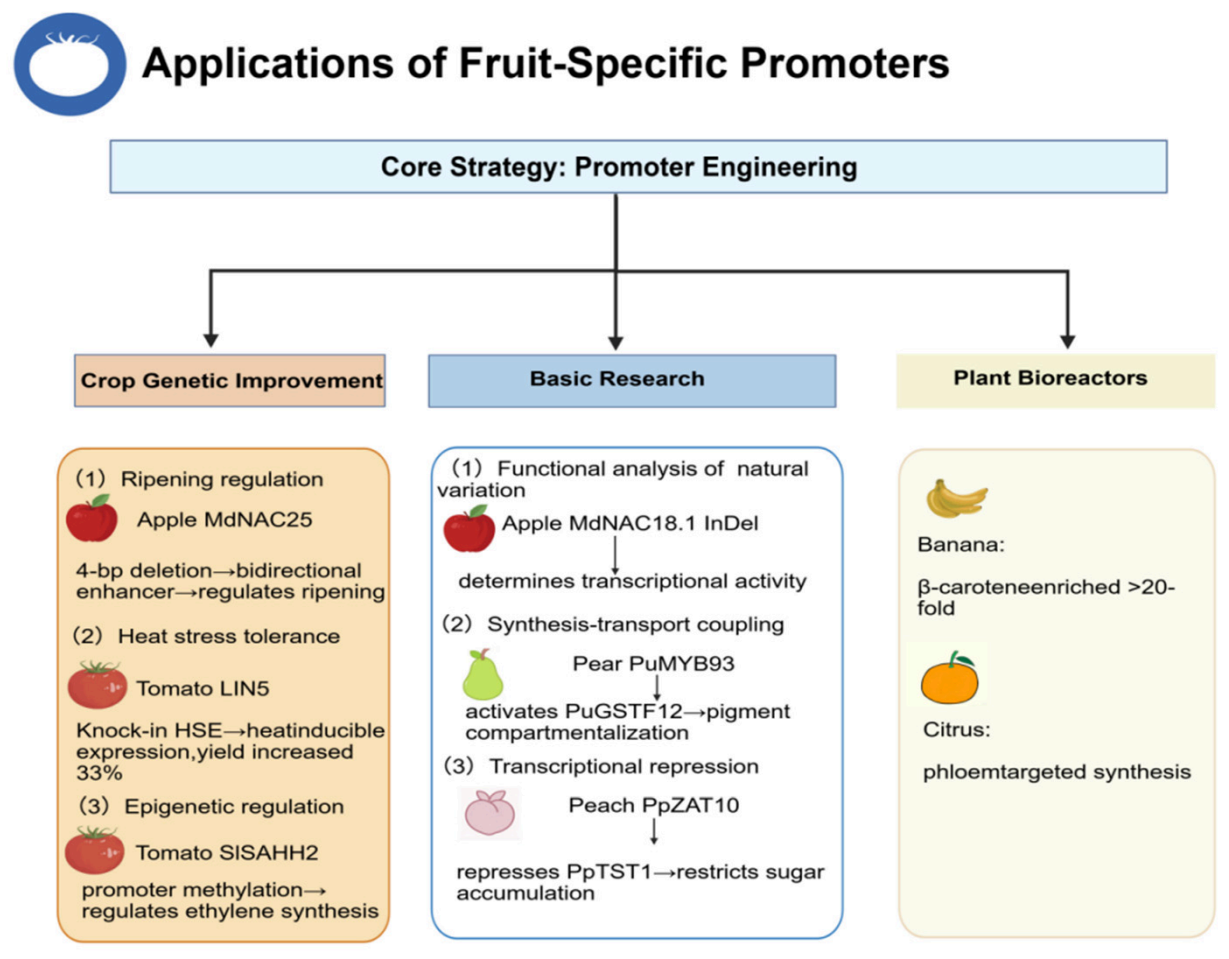
The Diverse Applications of Fruit-specific Promoters. (**Left**) (Crop genetic improvement): Applications include ripening regulation (apple MdNAC25), heat-stress tolerance (tomato LIN5 with knock-in HSEs, increasing yield by 33%), and epigenetic regulation (tomato SlSAHH2 promoter methylation). (**Middle**) (Basic research): Applications include functional analysis of natural promoter variants (apple MdNAC18.1 InDel), synthesis-transport coupling (pear PuMYB93-PuGSTF12 module), and transcriptional repression (peach PpZAT10-PpTST1). (**Right**) (Plant bioreactors): Examples include β-carotene biofortification in banana (>20-fold increase) and phloem-targeted synthesis in citrus. In the figure, the large black arrows indicate that promoter engineering diverges into three major application areas, while the various small black arrows represent the causal regulatory pathways by which upstream genes regulate downstream genes to ultimately produce the corresponding fruit traits. Created with BioGDP.com [168].

For example, in studies investigating the regulation of fruit ripening time in apples, researchers identified a 4 bp deletion variant within the *MdNAC25* promoter region. This deletion generated the ACS2-TAAAATAT cis-regulatory element, which not only enhanced *MdNAC25* expression but also functioned as a bidirectional enhancer to simultaneously activate the upstream antisense-transcribed *lncRNA-nac25*, thereby establishing an “*lncRNA–NAC* transcription factor” synergistic regulatory module (Figure 5). Population genetic analysis revealed that the frequency of the allele carrying this 4 bp deletion was significantly higher in cultivated apple varieties (33%) than in wild apple species (11%), suggesting that this variation may have undergone selection during domestication for early-maturity traits. Furthermore, heterologous expression of *MdNAC25* in the tomato nor mutant restored the expression of ripening-related genes and increased carotenoid accumulation threefold. This study revealed a novel regulatory mechanism in which natural promoter variation modulates fruit ripening through non-coding RNA-mediated regulation, providing potential targets for the precise control of fruit maturation timing [126] (Figure 5).

Luo et al. [186] developed the CROCS (Climate-Responsive Optimization of Carbon Partitioning to Sink) strategy based on prime editing, enabling the precise insertion of heat shock elements (HSEs) into the promoter region of cell wall invertase genes (*CWINs*). The study demonstrated that under heat stress conditions, downregulation of *LIN5* (encoding *CWIN*) expression is a major factor contributing to impaired fruit development and reduced yield in tomato. By introducing a 10 bp heat-responsive element into the *LIN5* promoter, the researchers successfully converted *LIN5* into a heat-inducible gene. Under field heat stress conditions, the edited tomato plants exhibited increased fruit set and single fruit weight, resulting in a 33% increase in yield per plot compared with heat-stressed controls, without negatively affecting fruit quality parameters such as soluble solids content (Brix value).

Yang et al. [187] demonstrated that the tomato *SlSAHH2* gene influences fruit ripening by maintaining DNA methylation homeostasis. CRISPR-mediated knockout of *SlSAHH2* resulted in delayed fruit ripening, accompanied by increased methylation levels in the promoter regions of ethylene biosynthesis-related genes, including *E4*, *E8*, *ACO1*, *ACO3*, and *ACS2*. Correspondingly, the expression levels of these genes were generally reduced, highlighting the important role of epigenetic regulation in controlling fruit ripening-associated gene expression.

Compared with conventional strategies involving transgenic overexpression or gene knockout, promoter engineering provides several advantages. It enables enhancement or fine-tuning of endogenous gene expression without modifying protein-coding sequences, thereby minimizing potential pleiotropic effects (Figure 5). Moreover, targeted editing of conserved cis-regulatory elements provides opportunities for cross-species trait improvement and the generation of “non-transgenic” breeding materials without foreign DNA integration. Such approaches not only facilitate the improvement of agronomic traits but also enhance the potential for industrial deployment and regulatory acceptance. Therefore, promoter engineering is increasingly recognized as an important strategy for precision breeding in crop improvement.

### 5.2. Application in Plant Bioreactors

In plant biotechnology, the term “plant bioreactor” refers to the large-scale production of biologically valuable compounds, including proteins and metabolites, using transgenic plants as bio-production platforms. This approach encompasses the production of plant-, human-, or animal-derived proteins, peptides, and essential amino acids; more broadly, it involves the utilization of plant cells or tissues for the synthesis of pharmaceutical compounds with medicinal value or important biological functions. The choice of promoter is a critical determinant of target product yield, as well as the efficiency and complexity of subsequent extraction and purification processes. Strong constitutive promoters, such as the *CaMV 35S* promoter and ubiquitin promoters, can drive high-level expression of target genes (Figure 5), thereby increasing product accumulation.

Oussama et al. [188] utilized tomato as a plant bioreactor and employed fruit-specific promoters to drive the high-level expression of key genes involved in the saffron apocarotenoid biosynthetic pathway, including *CsCCD2L*, *CsUGT2*, and *UGT709G1*, specifically in tomato fruits. This strategy enabled the heterologous and efficient biosynthesis of high-value saffron-derived apocarotenoids, including crocin and picrocrocin. The engineered tomato fruits accumulated crocin at levels of up to 14.48 mg/g dry weight and picrocrocin at 2.92 mg/g dry weight. These levels represent the highest yields reported for heterologous metabolic engineering of these compounds. Moreover, extracts from the engineered fruits exhibited significant antioxidant activity and neuroprotective potential.

Tindamanyire et al. [189] employed banana (*Musa* spp., *Cavendish variety*) as a plant bioreactor by utilizing fruit-specific promoters, such as the BananaExpansin1 and patatin-like promoters, to drive fruit-specific expression of key carotenoid biosynthetic genes derived from banana or maize (*PaCrtI*, *ZmPSY*, etc.). This strategy successfully generated biofortified bananas with substantially enhanced provitamin A (β-carotene) accumulation. Through Agrobacterium-mediated genetic transformation, multiple transgenes were introduced and stacked in the banana genome. Multi-year, multi-location field trials demonstrated that the engineered traits were stably maintained during fruit ripening, with β-carotene levels increasing by more than 20-fold compared with wild-type plants. The enhanced carotenoid phenotype was stably inherited over three consecutive generations, while no significant differences in major fruit agronomic traits were observed between transgenic and control plants. This study provides important field-based evidence supporting the potential commercial application of fruit-specific promoter-driven bioreactor systems for nutritional biofortification.

Bezerra et al. [173] used citrus as a model system to systematically evaluate the potential of multiple endogenous phloem-preferential promoters, including *CsPP2*, *CsSUT1*, and members of the *CsPHLOEM* family, for driving transgene expression through Agrobacterium-mediated genetic transformation. The aim was to develop plant bioreactor systems capable of producing secondary metabolites in phloem tissues. Although these promoters primarily exhibited phloem-specific expression patterns, the study further demonstrated that optimized promoter elements could drive the expression of carotenoid biosynthetic genes (*CsPSY*, *CsBCH*) in the phloem and associated vascular tissues of tomato fruits, resulting in targeted accumulation of carotenoid metabolites in fruit vascular regions. This work provides valuable tissue-specific promoter resources and strategic insights for exploiting fruit vascular tissues as specialized bioreactor platforms.

### 5.3. Application in Basic Molecular Biology Research

Fruit-specific promoters are valuable tools for dissecting gene functions and regulatory networks, with broad applications in basic plant research. By fusing target genes with fruit-specific promoters, gene expression can be precisely controlled in a tissue-specific manner, enabling detailed functional characterization of target genes (Figure 5).

For example, Yue et al. [62] demonstrated through association analysis and genetic transformation that an InDel variation in the promoter region of the apple *NAC* transcription factor *MdNAC18.1* is a key determinant of its transcriptional activity. By driving *MdNAC18.1* expression specifically in tomato fruits using its promoter, the study revealed that *MdNAC18.1* functions as a central regulator within the fruit ripening network by modulating downstream genes involved in ethylene biosynthesis.

Anthocyanin-mediated fruit coloration depends not only on the efficiency of anthocyanin biosynthesis but also, critically, on the transport and sequestration of anthocyanins into vacuoles. Sun et al. [190] employed a pear fruit transient expression system to demonstrate that the transcription factor *PuMYB93* does not directly regulate anthocyanin structural genes; instead, it specifically activates the downstream transporter gene *PuGSTF12*, thereby directing metabolic flux from anthocyanin biosynthesis toward vacuolar accumulation. This study highlights the unique utility of fruit-specific promoters in elucidating the coordination between anthocyanin synthesis and transport, demonstrating how tissue-specific promoter activity can facilitate the dissection of metabolic compartmentalization mechanisms.

Sugar accumulation in fruits exhibits strong spatiotemporal regulation and is initiated within fruit tissues at specific developmental stages. Liu et al. [191] exploited this characteristic to investigate the negative regulatory role of the transcription factor *PpZAT10* on the vacuolar sugar transporter gene *PpTST1* in peach fruits using a transient expression system. The study showed that *PpZAT10* specifically binds to cis-regulatory elements within the *PpTST1* promoter and represses its transcription during fruit ripening, thereby restricting the extent of sugar accumulation. By using endogenous fruit regulatory elements as a functional framework, this study revealed how transcriptional repression contributes to the precise spatial and temporal control of sugar accumulation during fruit quality formation.

## 6. Conclusions and Future Perspectives

**Figure 6:**
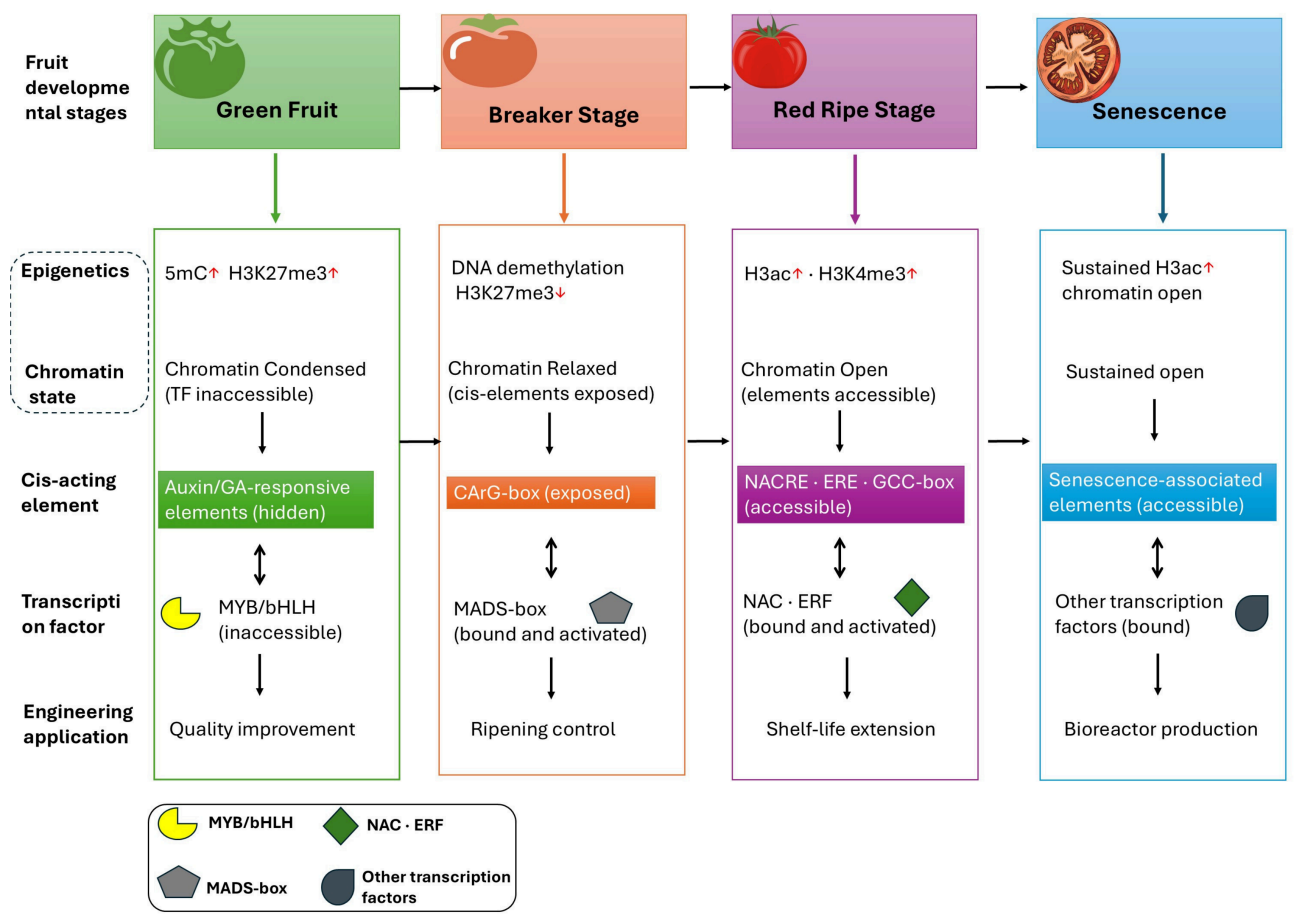
Conceptual framework integrating epigenetic regulation, cis-regulatory architecture, transcription factor networks, and engineering applications across fruit developmental stages. The model is organized into four columns representing Green Fruit, Breaker, Red Ripe, and Senescence. Within each column, five interconnected layers are arranged vertically: epigenetic marks and chromatin state, cis-regulatory elements, transcription factor families, and engineering applications. Horizontal arrows (→) indicate developmental progression; vertical arrows (↓) indicate causal relationships; bidirectional arrows (↕) indicate direct TF–cis-element correspondences. Parenthetical annotations indicate the functional status at each stage. Additionally, red upward-pointing arrows indicate an increase, and red downward-pointing arrows indicate a decrease.

Based on the systematic review of fruit-specific promoter classification, regulatory mechanisms, research methodologies, and applications presented above, we propose an integrated conceptual framework (Figure 6) that places cis-regulatory architecture, transcription factor networks, epigenetic regulation, and engineering applications within a unified developmental timeline and hierarchical regulatory context.

As fundamental regulators of gene expression, fruit-specific promoters play essential roles in plant molecular biology and crop improvement. Over several decades, research in this field has progressed from the isolation and characterization of individual promoters toward a comprehensive understanding of regulatory networks, synthetic promoter design, and precision genome engineering. Despite these substantial advances, significant challenges remain, while emerging technologies continue to provide new opportunities for future development.

### 6.1. Current Research Status and Challenges

Although numerous fruit-specific promoters have been cloned and functionally characterized, and considerable progress has been made in elucidating the interactions between cis-regulatory elements and transcription factors, several fundamental questions remain unresolved. First, the synergistic regulation mediated by multiple cis-regulatory elements within fruit-specific promoters remains poorly understood. The interactions and hierarchical relationships among different regulatory modules, including enhancers, silencers, and core promoter regions, during fruit development and environmental adaptation require further systematic investigation. Second, the contribution of three-dimensional (3D) chromatin architecture to fruit-specific promoter regulation remains largely unexplored. How chromatin folding and spatial interactions between promoters and distal regulatory elements contribute to the spatiotemporal control of gene expression during fruit development remains an important unanswered question. Third, the functional interplay between epigenetic modifications, including DNA methylation, histone modifications, and non-coding RNA regulation, and cis-regulatory elements requires further clarification. In particular, the dynamic changes and regulatory consequences of epigenetic modifications on promoter activity throughout fruit development, ripening, and stress responses remain to be fully characterized.

#### 6.1.1. Technical Limitations in Promoter Characterization

Despite significant advances in promoter cloning, functional characterization, and regulatory network analysis, several technical limitations continue to restrict the efficiency and depth of fruit-specific promoter research. A major challenge is the generation of stable transgenic materials in fruit crops, particularly woody species such as apple, citrus, and pear. Stable transformation in these species remains highly time-consuming and technically demanding, with the production of transgenic plants often requiring several years. This limitation substantially delays promoter functional validation and the dissection of underlying regulatory mechanisms. Another limitation arises from the widespread use of heterologous expression systems. Model systems, including tobacco and tomato, are frequently employed for promoter activity evaluation; however, they may not fully recapitulate the endogenous regulatory environment of the original fruit species. Differences in transcription factor repertoires, chromatin accessibility, and epigenetic landscapes may lead to discrepancies between promoter activity observed in heterologous systems and its native expression patterns, thereby affecting the accuracy and reliability of functional characterization. Furthermore, current approaches remain insufficient for resolving the complex regulatory networks controlled by fruit-specific promoters. Existing technologies have limited capacity to comprehensively analyze interactions among multiple promoters, transcription factors, and downstream target genes, which restricts a systematic understanding of promoter-mediated transcriptional regulation.

#### 6.1.2. Challenges in Practical Applications of Fruit-Specific Promoters

At the application level, the utilization of fruit-specific promoters in crop improvement and plant bioreactor systems still faces several important challenges. On the one hand, naturally occurring fruit-specific promoters have inherent limitations. Many native promoters exhibit relatively weak transcriptional activity compared with strong constitutive promoters, limiting their suitability for high-level production of target compounds, such as pharmaceutical proteins and valuable metabolites. In addition, some promoters display incomplete tissue specificity, resulting in detectable ectopic expression in vegetative tissues and potential unintended effects on plant growth and development. Furthermore, the limited conservation of promoter regulatory mechanisms among different species restricts their direct application across diverse fruit crops. On the other hand, synthetic promoter development remains constrained by the lack of predictive design principles and robust engineering frameworks. Current synthetic promoter construction strategies largely rely on the combination of previously characterized cis-elements, whereas accurate prediction of expression strength, specificity, and stability remains challenging. Consequently, the efficiency and reliability of synthetic promoter development require further improvement. Moreover, commercialization of promoter-engineered crops is influenced by biosafety regulations and public acceptance. Potential environmental and food safety concerns associated with genetically modified crops containing engineered regulatory elements remain important considerations. In addition, differences in regulatory frameworks among countries and regions continue to create barriers to the industrial deployment of promoter engineering technologies.

#### 6.1.3. Limitations of Native Fruit-Specific Promoters

Despite their value as endogenous regulatory elements, native fruit-specific promoters still exhibit several intrinsic limitations that restrict their widespread application in crop improvement. A primary limitation is their relatively low transcriptional activity. The expression strength of most native fruit-specific promoters is considerably lower than that of strong constitutive promoters, such as the *CaMV 35S* promoter. Kim et al. demonstrated that a synthetic promoter composed of three tandem repeats of the *SlHDC-A* core-ES exhibited higher activity than the *CaMV 35S* promoter in transgenic tomato fruits at the orange stage, indirectly emphasizing the limited driving capacity of many native promoters [86]. Another major challenge is insufficient tissue specificity. Dasgupta et al. reported that among the citrus and plum promoters analyzed, only *CitSEP* displayed strict fruit-specific activity, whereas *CitVO1*, *CitUNK*, and *PamMybA* promoters exhibited weak but detectable expression in leaves and other vegetative tissues [55]. Furthermore, the species-specific nature of native promoters greatly restricts their cross-species applicability. Agius et al. found that the strawberry *GalUR* promoter showed no detectable activity in transiently transformed ripe tomato fruits, indicating that its regulatory function relies on species-specific transcriptional networks and regulatory contexts within strawberry fruit tissues [192]. Therefore, native promoters isolated from one species cannot be directly assumed to function effectively in other crops. Species-specific screening and functional validation remain necessary, which substantially increases the time, cost, and complexity of promoter development.

#### 6.1.4. Biases Associated with Heterologous Expression Systems

The limitations of heterologous validation systems represent another important consideration in fruit-specific promoter research. Currently, promoters isolated from non-tomato fruit species are frequently evaluated using model systems such as tomato or tobacco. However, these heterologous systems may not accurately reflect the endogenous regulatory environment of the original species, including differences in transcription factor repertoires, chromatin states, and epigenetic modifications. Agius et al. employed a strawberry fruit transient transformation system to evaluate the tomato polygalacturonase promoter and the pepper fibrillin promoter. They found that the *PG* promoter exhibited no significant activity in strawberry fruits, whereas the strawberry GalUR promoter showed no detectable activity in ripe tomato fruits [192]. These findings demonstrate that promoter activity may be highly dependent on species-specific regulatory contexts. Therefore, establishing efficient homologous transformation systems and validating promoter activity within native species represent critical strategies for improving the accuracy of promoter characterization.

#### 6.1.5. Challenges in Synthetic Promoter Design

The rational design of synthetic promoters also remains a major challenge. Nayak et al. emphasized that the application of natural regulatory modules in synthetic circuits is limited by three major factors: incomplete understanding of complex plant regulatory networks, insufficient characterization of functional promoter elements, and potential interference with endogenous regulatory pathways [87]. Although the functions of individual cis-elements can often be experimentally determined, assembling these elements into synthetic promoters with predictable expression patterns remains difficult. Furthermore, most current synthetic promoter designs rely primarily on simple combinations of known regulatory elements, without fully considering synergistic interactions among elements, optimal spacing, or chromatin context. These challenges highlight the need to integrate rational design approaches with high-throughput screening and machine-learning-based prediction strategies to develop more predictable and efficient synthetic promoters.

#### 6.1.6. Biosafety and Regulatory Challenges for Commercial Applications

Despite the promising potential of fruit-specific promoter-based technologies in crop improvement and plant biomanufacturing, their commercial deployment remains subject to several biosafety and regulatory considerations. One major concern is food safety. Although fruit-specific promoters can restrict transgene expression to edible tissues, the accumulation of foreign proteins or metabolites in fruits requires comprehensive assessment, including evaluations of potential toxicity, allergenicity, and nutritional impacts. Environmental risks represent another important consideration. Transgenic crops containing promoter-driven expression cassettes may potentially facilitate the movement of introduced genetic materials into wild relatives through pollen-mediated gene flow, necessitating careful ecological risk assessment before large-scale cultivation. In addition, regulatory frameworks for genetically modified and genome-edited crops differ substantially among countries and regions, creating uncertainty for commercialization. Horticulture Research recently summarized the development background, potential benefits, limitations, and commercial prospects of transgenic and genome-edited fruit crops [193]. For example, Tropic’s non-browning banana has received regulatory approval or exemption in 11 countries, including the United States, Canada, Japan, and Brazil; however, the approval procedures, evaluation criteria, and timelines vary considerably among these markets, reflecting the lack of global regulatory harmonization [193]. Such regulatory disparities increase the cost, complexity, and uncertainty associated with the commercialization of fruit crops developed through promoter engineering.

### 6.2. Future Development Directions

#### 6.2.1. Basic Research: Toward Precision and Systematic Exploration

Future basic research on fruit-specific promoters will increasingly focus on achieving higher-resolution and more systematic understanding of promoter function to address current knowledge gaps. A major direction is the application of single-cell resolution approaches to precisely characterize the spatiotemporal activity patterns of fruit-specific promoters across distinct cell types, including juice sacs, vascular bundles, and epidermal cells, throughout fruit development and stress responses. Such approaches will provide new insights into cell-type-specific regulatory mechanisms underlying fruit-specific gene expression. Another important research frontier involves elucidating the relationship between three-dimensional (3D) genome organization and promoter activity. Particular attention should be given to understanding how chromatin looping and spatial interactions between promoters and distal regulatory elements, such as enhancers, contribute to the hierarchical regulation of fruit-specific gene expression. In parallel, systematic identification of conserved regulatory elements across fruit crop species will facilitate the discovery of core cis-acting modules with broad applicability. Comparative analyses among diverse fruit crops, including members of the Rosaceae, Rutaceae, and Musaceae families, may provide valuable resources for developing transferable promoter systems across species.

Furthermore, artificial intelligence (AI)-assisted prediction and design of regulatory elements represent a promising avenue for future promoter engineering. By integrating multi-omics datasets, including genomic, transcriptomic, and epigenomic information, predictive models with improved accuracy could be established to characterize cis-elements and transcription factor binding patterns, ultimately enabling the rational design of promoters with predefined expression profiles.

#### 6.2.2. Technological Innovation: Overcoming Bottlenecks to Enhance Research Efficiency

Technological advances will be essential for overcoming current limitations in promoter discovery, characterization, and engineering. High-throughput promoter activity screening platforms based on microfluidics, cell-based systems, or transient expression approaches represent promising strategies for rapidly evaluating thousands of promoter fragments or variants, substantially reducing the time required for promoter identification and functional characterization. Meanwhile, the development of high-resolution technologies for mapping promoter–transcription factor interactions in vivo, including single-molecule ChIP-seq and live-cell imaging approaches, will enable dynamic monitoring of transcription factor binding to cis-regulatory elements within living fruit cells. In addition, further optimization of precise genome-editing technologies for in situ modification of cis-regulatory elements will provide powerful tools for promoter engineering. The integration of prime editing and base editing approaches may allow targeted modification of promoter sequences without disrupting endogenous coding regions, thereby enabling fine-scale control of promoter strength, specificity, and responsiveness.

#### 6.2.3. Application Expansion: Targeted Innovation for Horticultural and Agricultural Needs

##### Horticultural Breeding and Agronomic Technology Improvement

In horticultural crop improvement, future applications of fruit-specific promoters will focus on developing coordinated multi-gene expression systems capable of simultaneously and precisely regulating multiple quality-associated traits, including fruit color, flavor, texture, and nutritional composition. Such strategies may help overcome undesirable genetic trade-offs among complex agronomic traits. Moreover, environmentally responsive smart promoters, such as heat, cold, or drought-inducible fruit-specific promoters, represent a promising strategy for achieving conditional regulation of gene expression under adverse environmental conditions while maintaining fruit quality. In parallel, the development of subcellular compartment-specific promoters targeting organelles or cellular compartments, including vacuoles, chloroplasts, and the endoplasmic reticulum, may facilitate precise localization of target proteins or metabolites, thereby enhancing the accumulation of valuable compounds while minimizing metabolic burdens.

##### Food Deep Processing and Functional Product Development

In food biotechnology, fruit-specific promoters are expected to provide powerful tools for establishing fruit-based plant bioreactors aimed at the efficient production of high-value functional compounds, including anthocyanins, carotenoids, bioactive peptides, and food-grade enzymes. These approaches may promote the development of functional fruit products, such as nutritionally enhanced fruits, natural food additives, and bioactive extracts, thereby extending the value chain of the fruit processing industry. Furthermore, precise regulation of key genes involved in fruit ripening and senescence pathways through fruit-specific promoters may contribute to extending postharvest shelf life, reducing storage losses, and improving the quality stability of processed fruit products.

#### 6.2.4. Industrialization and Standardization: Promoting Healthy and Orderly Development

To facilitate the industrial translation of fruit-specific promoter technologies, coordinated efforts are required in several key areas. One priority is the establishment of standardized frameworks, including guidelines for promoter source documentation, safety assessment procedures for promoter-driven products, and unified criteria for promoter functional validation. Such standardization will improve the reliability, reproducibility, and safety evaluation of promoter engineering applications. In addition, the development of open-access and integrated databases for fruit-specific promoters will provide valuable resources for researchers and breeders. These databases should incorporate promoter sequences, regulatory elements, functional characteristics, and representative application cases, thereby facilitating efficient resource utilization and knowledge sharing. Furthermore, enhanced collaboration among academic institutions, industry stakeholders, and regulatory agencies will be essential for addressing biosafety concerns, refining regulatory frameworks for genetically modified products, and accelerating the commercialization of promoter-engineered fruit varieties and plant-based bioreactor systems.

#### 6.2.5. General Rules for Fruit-Specific Promoter Design

Based on the systematic analysis of regulatory mechanisms, research methodologies, and engineering applications of fruit-specific promoters, several general principles for promoter design can be summarized as follows.

##### Promoter Design for Yield Improvement

Improving fruit yield generally requires enhancing sink strength or promoting cell proliferation and expansion. Promoter design strategies should therefore prioritize regulatory elements that drive strong expression during early fruit development, particularly during stages associated with cell division and expansion. Promoters exhibiting activity in green fruits or throughout fruit development may be particularly valuable for this purpose. Enhancing transcriptional strength through tandem duplication of key enhancer elements represents another potential strategy. For example, editing conserved cis-regulatory elements within the tomato *KLUH* promoter significantly increased fruit weight, demonstrating the feasibility of promoter engineering for yield improvement [142]. In addition, promoter selection should consider regulatory elements with strong activity in fruit sink tissues to maximize resource allocation toward developing fruits.

##### Promoter Design for Stress Tolerance Improvement

Enhancing stress tolerance requires promoters capable of rapidly responding to specific environmental stimuli. One effective strategy is the incorporation of stress-responsive cis-elements, such as heat shock elements (HSEs), drought-responsive elements (DREs), and low-temperature-responsive elements (LTRs), into promoter architectures upstream of core regulatory regions [4]. In tomato, the CROCS strategy employed prime editing to precisely introduce *HSE* motifs into the *LIN5* promoter, thereby conferring heat-inducible expression of a cell wall invertase gene and resulting in a 33% increase in yield under heat stress conditions [186]. This example highlights the potential of environmentally responsive promoter engineering for improving crop resilience.

##### Promoter Design for Ripening Control

Precise regulation of fruit ripening requires controlled modulation of genes involved in ethylene biosynthesis, signaling pathways, and downstream ripening processes. Ripening-associated promoters, such as *E8* and *PG* promoters, can be utilized to drive the expression of ethylene-related genes or ripening regulators [32]. Furthermore, promoter engineering provides opportunities to fine-tune the timing and intensity of ripening-associated gene expression. Dual-stage promoters, such as *2A11*, which remain active during both green fruit development and ripening stages, may be particularly useful for applications requiring regulation across multiple developmental phases [40].

##### Promoter Design for Metabolic Engineering

Metabolic engineering requires promoters capable of coordinating the expression of multiple genes within complex biosynthetic pathways. Fruit-specific promoters can be employed to drive key metabolic enzymes, whereas synthetic promoters assembled from multiple cis-regulatory modules may enable coordinated pathway regulation [4].

For example, fruit-specific promoters have been successfully used to drive genes involved in saffron apocarotenoid biosynthesis in tomato, resulting in high accumulation of crocin and picrocrocin [188]. However, promoter design for metabolic engineering should consider substrate availability, pathway balance, and potential metabolic burdens caused by excessive accumulation of intermediate metabolites.

##### Promoter Haplotype Considerations

Natural variation within promoter regions, including single nucleotide polymorphisms (SNPs) and insertion/deletion (InDel) variants, can substantially influence transcription factor binding affinity and promoter activity [4]. In apple, an InDel variant in the MdNAC18.1 promoter determines transcriptional activity and affects fruit ripening time [62]. Similarly, a 4 bp InDel variant in the *MdNAC25* promoter functions as a bidirectional enhancer and has undergone enrichment during the selection of cultivated varieties [143]. Therefore, promoter design should consider naturally occurring high-activity haplotypes or introduce favorable promoter variants into elite cultivars through precise genome editing.

##### Environmental Responsiveness Considerations

The development of smart promoters requires the integration of environmental signal-responsive regulatory modules to achieve precise spatiotemporal control of gene expression. Incorporating cis-elements responsive to environmental factors, including temperature, light, and water availability, represents an effective approach for constructing environmentally adaptive promoters [186]. Moreover, combining multiple response elements may enable coordinated regulation under complex environmental conditions [4]. The tomato CROCS strategy provides a successful example of applying heat-responsive promoter engineering to improve climate adaptability and yield stability [186].

##### Regulatory Approval Considerations

The design of promoters for commercial applications must also account for regulatory requirements. One important strategy is to prioritize endogenous promoter elements to minimize the introduction of foreign DNA sequences [4]. Precise editing of endogenous promoters, rather than insertion of exogenous expression cassettes, provides an opportunity to generate improved crops without foreign DNA integration, thereby potentially simplifying regulatory assessment [142,186]. In addition, promoter-driven products must satisfy food safety requirements, and promoter elements with well-characterized origins and functions should be preferentially selected to facilitate regulatory approval processes [193].

### 6.3. Conclusions

As fundamental regulators of gene expression, fruit-specific promoters are undergoing a significant transition from “empirical exploration” toward “rational design and precise engineering.” Over the past three decades, extensive research has generated valuable promoter resources, advanced our understanding of regulatory mechanisms, and established powerful technologies for promoter characterization and manipulation. Looking ahead, the integration of emerging technologies, including single-cell sequencing, three-dimensional genomics, artificial intelligence, and precision genome editing, is expected to transform fruit-specific promoters from tools enabling simple “tissue-specific expression” into sophisticated regulatory systems capable of temporally, spatially, and quantitatively precise control of gene expression. These advances will not only provide powerful strategies for molecular breeding of fruit quality traits but also facilitate the development of efficient fruit-based plant bioreactors, thereby contributing to global food security, improved nutritional quality, and sustainable development of the fruit industry. Nevertheless, realizing the full potential of fruit-specific promoter technologies requires continued integration of fundamental discoveries, technological innovation, and translational applications. Strengthening interactions among these interconnected fields will be essential for advancing promoter research and enabling the next generation of precision breeding and biotechnology applications.

## Figures and Tables

**Table 1:** List of different fruit-specific promoters.

Promoter Name	Source Species	Transformed Species	Developmental Stage	Driven Gene	Reference
E4	*S. lycopersicum*	*S. lycopersicum*	Ripening-specific	*GUS*	[38]
E11	*S. lycopersicum*	*S. lycopersicum*	Ripening-specific	*GUS*	[63]
SLY-MIR167A	*S. lycopersicum*	*S. lycopersicum*	Ripening-specific	*GUS*	[64]
PG	*S. lycopersicum*	*S. lycopersicum*	Ripening-specific	*IGF-1*	[65]
2A11	*S. lycopersicum*	*S. lycopersicum*	Dual-stage	*IGF-1*	[65]
TFM7	*S. lycopersicum*	*A. thaliana*	Green-fruit-specific	*AtCKX2*	[66]
Strawberry APX gene promoter	*F.×ananassa*	*F.×ananassa*	Ripening-specific	*GUS*	[67]
Apple ACC oxidase promoter	*M. domestica*	*S. lycopersicum*	Ripening-specific	*GUS*	[59]
2A12	*S. lycopersicum*	*S. lycopersicum*	Ripening-specific	*IPR*, *GUS*	[68]
AGPL1	*Citrullus lanatus*	*S. lycopersicum*	Green-fruit-specific	*G* *US*	[69]
FBP7	*Petunia ×hybrida*	*F.×ananassa*	Dual-stage	—	[70]
P119	*S. lycopersicum*	*F.×ananassa*	Dual-stage	*PL*	[36]
B33	*Solanum tuberosum*	*A. thaliana*	Tuber-specific	*GUS*	[71]
MNACO	*Morus notabilis*	*A. thaliana*	Ripening-specific	*GUS*	[72]
CMACOI	*Cucumis melo*	*Cucumis melo*	Ripening-specific	*GUS*	[73]
E8	*S. lycopersicum*	*S. lycopersicum*	Ripening-specific	*MONELLIN*	[74]
EEF48	*Solanum melongena*	*Solanum melongena*	Ripening-specific	*CRTB*	[75]
SIPPC2	*S. lycopersicum*	*S. lycopersicum*	Green-fruit-specific	—	[76]
FVKNOX	*F.×ananassa*	*F.×ananassa*	Green-fruit-specific	*FVSTP* *8*	[77]
SlHDC-A	*S. lycopersicum*	*S. lycopersicum*	Fruit-specific	*G* *US*	[46]
SLADH2	*S. lycopersicum*	*S. lycopersicum*	Dual-stage	*G* *US*	[43]
RLP1	*S. lycopersicum*	*S. lycopersicum, A. thaliana*	Ripening-specific	*GUS*	[32]
ACS4	*S. lycopersicum*	*S. lycopersicum*	Ripening-specific	*GUS*	[78]
SLPR10	*S. lycopersicum*	*S. lycopersicum*	Fruit-specific	*GFP-GUS*	[79]
CITSEP	*Citrus* sp.	*S. lycopersicum*	Ripening-specific	*GUS*	[31]
CITWAX	*Citrus* sp.	*S. lycopersicum*	Fruit-preferential	*GUS*	[31]
CITJUSAC	*Citrus* sp.	*S. lycopersicum*	Fruit-preferential	*GUS*	[31]
CITVO1	*Citrus* sp.	*S. lycopersicum*	Fruit-preferential	*GUS*	[31]
GSX7R	*O. sativa*	*O. sativa*	Green-tissue-specific	*GUS*	[80]
ARASSU	*A. thaliana*	*Cicer arietinum*	Green-tissue-specific	*CRY2AA*	[81]
PLE	*S. lycopersicum*	*S. lycopersicum*	Green-tissue-specific	*GUS*, *GFP*	[25]
PNH	*S. lycopersicum*	*S. lycopersicum*	Whole-development	*GUS*, *GFP*	[25]
PSN	*S. lycopersicum*	*S. lycopersicum*	Whole-development	*GUS*, *GFP*	[25]
PHD	*S. lycopersicum*	*S. lycopersicum*	Whole-development	*GUS*, *GFP*	[25]
PFF	*S. lycopersicum*	*S. lycopersicum*	Dual-stage	*GUS*, *GFP*	[25]
LA22CD07	*S. lycopersicum*	*S. lycopersicum*	Whole-development	*GUS*	[33]
LesAffx.6852.1.S1_at	*S. lycopersicum*	*S. lycopersicum*	Whole-development	*GUS*	[33]
MDRBCS	*M. domestica*	*Nicotiana tabacum*	Green-tissue-specific	*GUS*	[52]
VVADH1	*V. vinifera*	—	Green-tissue-specific	—	[51]
VVADH2	*V. vinifera*	—	Ripening-specific	—	[51]
VVADH4	*V. vinifera*	—	Ripening-specific	—	[51]
MAEXP1	*M. acuminata*	—	Ripening-specific	—	[56]

**Table 2:** Common cis-regulatory elements in fruit-specific promoters and their functions.

Element Type	Element Name	Species Discovery	Core Sequence	Primary Function	Reference
Core Promoter Elements	CAAT-box	—	CAAT	Basic promoter element; influences transcription initiation efficiency	[5]
	TATA-box	—	TATA	Basic promoter element; determines transcription start site	[6]
Hormone-Responsive Elements	ABRE	*C. papaya*	CACGTG	Responsive to ABA signaling	[18]
	GARE-motif	*P. persica*	TCTGTTG	Responsive to GA signaling	[97]
	TATC-box	*P. persica*	TATCCCA	Responsive to GA signaling	[97]
	P-box	*P. persica*	CCTTTTG	Responsive to GA signaling	[97]
	TGA-element	*P. persica*	AACGAC	Responsive to IAA signaling	[97]
	TGACG-motif	*P. persica*	TGACG	Responsive toMeJA signaling	[97]
	CGTCA-motif	*P. persica*	CGTCA	Responsive to MeJA signaling	[97]
	AuxRR-core	*Nicotiana tabacum*	GGTCCAT	Responsive to IAA signaling	[98]
	TCA	*Brassica oleracea*	GAGAAGAATA	Responsive to SA signaling	[99]
Ethylene-Responsive Elements	ERE	*S* *. lycopersicum*	ATTTTAAA	Ethylene-responsive element; plays a key role in fruit ripening	[100]
	GCC-box	*Nicotiana*, *S. lycopersicum*	AGCCGCC	Ethylene-responsive element; plays a key role in fruit ripening	[101]
Light-Responsive Elements	G-Box	*Zea mays*	CACGTT	bHLH transcription factor binding site; responsive to light and stress	[102]
	GT1-box	*F.×ananassa*	GGTTAA	Recognized by GT-1 factor; involved in light-regulated gene expression	[98]
	I-box	*F.×ananassa*	GATAA	MYB transcription factor binding site; regulates flavonoid biosynthesis and defense	[98]
	Box 4	*Petroselinum crispum*	ATTAAT	Involved in transcriptional regulation	[102]
	AE-box	—	AGAAACAA	Involved in transcriptional regulation	[102]
Stress/Defense-Responsive Elements	ARE	*Zea mays*	AAACCA	Anaerobic induction element	[98]
	MBS	*A. thaliana*	YAACKG	Drought-inducible element	[98]
	LTR	*Hordeum vulgare*	CCGAAA	Low-temperature responsive element	[103]
	W-Box	*A. thaliana*	GGTCAA/ TTGACC	WRKY factor binding site; responsive to pathogens and stress; regulates anthocyanin biosynthesis	[104]
	STRE	*Saccharomyces cerevisiae*	AGGGG	Involved in response to various stress conditions	[102]
Transcription Factor Binding Sites	MYB	—	CAACAG	MYB family binding site; regulates cell cycle and stress	[105]
	MYC	*A. thaliana*	TAACTG	MYC family binding site; involved in growth and development	[102]
	CArG box	*Homo sapiens*	CC(A/T)6GG	Specific binding site for *MADS*-box transcription factors	[106]

## Data Availability

Data are contained within the article.
